# From Automation-Induced Job Loss to a Supervisory Economy?

**DOI:** 10.12688/f1000research.168512.2

**Published:** 2026-02-11

**Authors:** Peter Malliaros, W Alejandro Pacheco-Jaramillo

**Affiliations:** 1 Research, UrCommunity Ltda., Melbourne, VIC, 3051, Australia; 2 Faculty of Business, Government and Law, University of Canberra, Canberra, ACT, 2617, Australia

**Keywords:** Artificial intelligence (O33); Automation (O33); Supervisory economy (J21); Labour-market dynamics (J21); Unemployment (E24); Human capital (J24); Technological change (O33)

## Abstract

**Background:**

Rapid advances in general-purpose artificial intelligence are compressing automation timelines: around 40–50% of tasks in advanced economies are technically automatable, and a quarter of total hours could be migrated to machines before 2030. Fears of large-scale displacement from artificial-intelligence (AI) adoption have prompted calls for either mass reskilling or unconditional cash transfers. Another option, the emergence of a “supervisory economy” in which humans specialise in overseeing AI systems, remains empirically under-examined.

**Methods:**

Using a balanced panel of 12 economies observed annually from 2014 to 2023, we construct a sector-weighted AI-exposure index and match it to labour-force data on unemployment, supervisory employment, public transfers, R&D, and GDP per capita. Two-way fixed-effects regressions are estimated, first linearly and then with a quadratic AI term to capture non-linearity in technology shocks. Robustness checks include lagged covariates, alternative normalisations and clustered standard errors.

**Results:**

The preferred quadratic specification reveals an inverted-U relationship between aggregate AI exposure and unemployment: joblessness rises at low-to-moderate exposure but falls once adoption surpasses approximately 1.7 standard deviations above the mean. Supervisory depth—measured as the share of senior- and middle-management roles—has no significant standalone effect and does not significantly moderate AI’s impact. Higher real income consistently dampens unemployment, while public transfers and contemporaneous R&D outlays show limited short-run cushioning.

**Conclusions:**

National labour markets appear to follow a concave AI trajectory: modest automation initially displaces routine labour, but at high penetration, complementary demand offsets job losses. However, simply expanding managerial layers is insufficient to buffer early shocks; effective resilience likely hinges on coupling oversight capacity with targeted technical upskilling and productivity-enhancing growth. Policy mixes that rely primarily on cash transfers or untargeted innovation spending risk delaying, rather than mitigating, employment re-equilibration in the age of AI.

## Introduction

Artificial intelligence (AI) is no longer a distant prospect, but a general-purpose technology that is reshaping labour demand in real-time. Seminal forecasts estimate that 40–50 per cent of current tasks in advanced economies are within the technical reach of automation, while successive World Economic Forum surveys predict that roughly one-quarter of all hours worked could be displaced by machines before 2030 ( Frey & Osborne, 2017; WEF, 2025). Empirical evidence already confirms the initial stages of this transition: in Europe and North America, mid-skill, routine-intensive occupations have contracted sharply, while employment has expanded simultaneously in low-skill personal services and high-skill analytical roles ( Autor, 2015; Goos et al., 2014). However, a second, quieter trend is emerging. Vacancy data from Australia, Germany and the United States show sustained growth in postings whose core duties are supervising, auditing or contextualising AI systems **—**positions labelled “AI-governance analyst”, “algorithmic-risk manager” or “quality-assurance lead”. These roles exploit human strengths in ethical judgement, situational awareness, and social trust, suggesting that a sizable share of labour displaced by automation might be reabsorbed as oversight labour rather than as direct substitutes for machines.

Existing policy prescriptions struggle to accommodate this possibility. Mass-retraining initiatives are often outpaced by rapid model cycles, and universal basic income (UBI) pilots in Finland and Kenya have improved self-reported well-being without increasing sustained employment ( Kangas et al., 2019; Banerjee et al., 2020). What remains missing is a systematic evaluation of whether an economy can pivot from doing to supervising—and whether such a pivot outperforms cash transfers on both employment and fiscal grounds.

As breakthroughs in artificial intelligence now outpace the slow churn of conventional up-skilling systems, redirecting displaced workers into AI-supervisory roles emerges as a more realistic safeguard of economic dignity than relying on training programmes that inevitably lag the technological frontier.

This paper fills that gap. Anchored in employment-polarisation theory ( Autor, Levy & Murnane, 2003), human-capital theory ( Becker, 1964) and economic-dependency theory ( Prebisch, 1950), we propose the concept of a Supervisory Society: a labour-market equilibrium in which humans specialise in oversight tasks that AI cannot internalise. Conceptually, the supervisory economy is not advanced here as a normative policy ideal nor as an inevitable stage of technological development. Rather, it is treated as a contingent labour-market configuration in which human oversight tasks expand as automation intensifies, conditional on institutional capacity, income levels, and complementary skills. Whether such a configuration emerges empirically is therefore an open question addressed in the analysis below. We test three hypotheses: (H1) sectors with high AI-adoption intensity and high automation risk experience disproportionate job loss; (H2) those same sectors, where institutional conditions permit, exhibit offsetting growth in supervisory vacancies; and (H3) UBI, while welfare-enhancing, does not restore employment at a comparable scale. We also control for leadership diversity by incorporating the female share of senior and middle management. This variable helps capture how gender-inclusive supervisory structures may enhance a country’s resilience to AI-driven labour market disruptions.

Methodologically, the study combines evidence at three levels. A balanced panel of 12 OECD and partner economies across 19 ISIC sectors (2015-2024) quantifies the *elasticity of AI risk* employment. High-frequency Internet Vacancy Index data for Australia (2015-2024) capture the emergence of oversight roles in real time, using triple-difference estimation around the 2020 diffusion of large language models. Finally, micro-panel data from the Finnish and Kenyan UBI experiments benchmark the employment effects and fiscal effort of unconditional transfers against the alternative of supervision.

By triangulating macro-sectoral displacement, meso-level vacancy creation, and micro-level income experiments, the paper offers the first integrated assessment of a controlled transition from traditional jobs to AI-supervised roles. The findings aim to inform governments seeking solutions that preserve dignity and purpose—behavioural factors known to condition public acceptance of automation, while avoiding the open-ended fiscal commitments implied by universal cash transfers.

## Literature review

Technological revolutions have consistently generated both profound anxiety and transformative economic progress throughout modern history. The canonical framework of *creative destruction*, as articulated by Schumpeter (1942), posits that technological innovation inherently dismantles established economic structures while simultaneously creating new industries and occupations. This dual process is evident in historical precedents: during Britain’s Industrial Revolution, agricultural employment plummeted from 80% to just 3% of the workforce, yet manufacturing absorbed displaced labourers through unprecedented productivity gains and the creation of new occupational categories ( Allen, 2009). Similarly, late 20th-century computerisation eradicated approximately 3.5 million routine clerical positions across OECD nations between 1980 and 2010 but concurrently generated 19 million new roles in cognitive services and non-automatable sectors ( Autor et al., 2003). These transitions validate economic theory’s long-term optimism regarding technological unemployment, as disruption is inevitable. However, compensation mechanisms have historically prevailed through demand expansion, occupational diversification, and productivity spillovers.

The contemporary artificial intelligence revolution, however, exhibits distinctive characteristics that challenge historical analogies. Unlike prior automation waves focused on manual or routine cognitive tasks, AI systems are increasingly demonstrating competence in domains that require advanced pattern recognition, contextual judgment, and creative synthesis—capabilities previously considered exclusively human ( Webb, 2020). Patent analysis reveals AI can currently perform 15% of tasks undertaken by top-quartile wage earners, including medical diagnostics, legal research, and algorithmic financial trading ( Webb, 2020). This cognitive breadth distinguishes AI from earlier technologies, threatening professional classes once insulated from automation. Moreover, the velocity of AI diffusion is unprecedented; generative tools like ChatGPT achieved 100 million users within two months, ten times faster than previous general-purpose technologies ( Our World in Data, 2023). Such acceleration compresses adaptation timelines for workers and institutions alike, potentially exacerbating displacement shocks.

Empirical evidence reveals divergent labour market impacts contingent on skill complementarity and institutional contexts. In OECD economies, occupations characterised by high computer usage exhibit 2.1% annual employment growth when integrated with AI, whereas low-digital-intensity occupations experience 1.7% reductions in working hours ( OECD, 2022). This bifurcation highlights AI’s dual role as both a productivity multiplier for digitally fluent workers and a substitute for others. Firm-level evidence from Taiwan indicates AI-adopting enterprises increased productivity by 14% while reducing non-AI hiring by 6.2%, suggesting task reorganisation rather than categorical job destruction ( Liang, 2024). Macroeconomic projections further support cautious optimism: the World Economic Forum (2020) anticipates 97 million AI-driven occupations emerging by 2025—primarily in machine learning, renewable energy, and care economies—offsetting 85 million displaced roles for a net global gain of 12 million jobs. Nevertheless, distributional consequences remain severe; workers lacking tertiary education face displacement probabilities three times higher than degree holders ( ILO, 2021).

The rapid advancement of artificial intelligence (AI) and automation technologies continues to fundamentally restructure global labour markets, generating profound concerns about employment sustainability and economic inequality. Frey and Osborne’s (2017) projection that 47% of U.S. employment faces a high automation risk has been substantiated cross-nationally through Organisation for Economic Co-operation and Development (OECD) studies, which show 14-32% job displacement probabilities across advanced economies ( Arntz et al., 2016). This technological disruption manifests as pronounced occupational polarisation, where employment growth concentrates at both skill extremes while mid-skill occupations experience disproportionate erosion ( Autor, 2015; Goos et al., 2014). Crucially, generative AI systems, such as large language models, have accelerated the displacement into cognitive domains previously considered resistant to automation. Recent analyses indicate that 80% of the U.S. workforce could see at least 10% of their tasks automated by generative AI, with roles involving writing, coding, and information processing facing 40-50% task exposure ( Eloundou et al., 2023; Felten et al., 2023). This represents a paradigm shift beyond traditional routineness-based displacement models, necessitating reconceptualisation of labour adaptation frameworks.

Profound distributional challenges nonetheless threaten to eclipse aggregate employment gains. Wage polarisation intensifies as AI-complementary workers command 17% premiums over those in substitutable roles ( Felten et al., 2019). Geographical disparities widen simultaneously; advanced economies exhibit 60% AI exposure, compared to 26% in low-income nations. However, developing regions face higher displacement risks due to occupational structures concentrated in automatable tasks and weaker social safety nets ( UNDP, 2023). Gender asymmetries compound these inequities, with women in OECD countries experiencing 9.6% job vulnerability compared to 3.5% among men—a disparity rooted in occupational segregation and digital access gaps ( ILO, 2021). These intersecting inequalities necessitate targeted policy innovations, including robot taxation schemes that fund portable reskilling accounts (modelled on Singapore’s SkillsFuture), strengthened collective bargaining frameworks, and ethical AI governance that prevents algorithmic discrimination.

Recent scholarship converges on the view that the tempo of artificial-intelligence breakthroughs now outstrips the cadence of formal up-skilling systems, creating a structural lag between the obsolescence of traditional tasks and workers’ ability to secure the competencies demanded by the digital economy. Frey and Osborne (2017) projected that nearly half of current jobs are technically automatable, yet vocational curricula remain locked into multi-year accreditation cycles. The gap has widened: laboratory estimates suggest that large language models such as GPT-4 can reconfigure up to 40 per cent of cognitive tasks within months of release ( Eloundou et al., 2023), leaving policymakers “running up a down escalator” ( Davenport & Kirby, 2016). Autor’s (2015) evidence shows that mid-skill jobs disappear faster than replacement roles can be generated, while human-capital theory implies that when the payoff horizon for new schooling shrinks, incentives to retrain erode ( Becker, 1964). Taken together, these dynamics reinforce this paper’s premise that pivoting displaced workers into AI-supervisory positions—where uniquely human judgement and ethical oversight remain indispensable—offers a more feasible absorption pathway than relying on up-skilling regimes that inevitably trail the technological frontier.

Despite extensive documentation of automation risks, significant knowledge gaps persist regarding sector-specific AI adoption trajectories and their precise relationship to the emergence of supervisory roles. Manufacturing automation, for instance, follows fundamentally different displacement patterns than service-sector AI implementation. Autor et al. (2022) demonstrate that healthcare and education automation generate 23% higher supervisory role demand than manufacturing, reflecting domain-specific requirements for human oversight in ethically sensitive contexts. This sectoral heterogeneity remains underexplored in current policy formulations, particularly concerning developing economies where labour market institutions face capacity constraints ( World Bank, 2023). Understanding these nuances is essential for designing targeted labour market interventions.

Because our dataset ends in 2023—precisely the year when generative AI adoption accelerates at an exponential pace—the assumption of a linear, constant-slope relationship between AI exposure and unemployment becomes untenable. Accordingly, imposing a linear constant-slope specification would be inconsistent with both recent diffusion evidence and established theories of technological adjustment, motivating the quadratic formulation adopted in the empirical section. Recent diffusion curves show that post-2023 advances in large language models and foundation-model tooling expand the scope of automatable tasks far more rapidly than prior, incremental waves of digitalisation. To accommodate this empirical reality, we enrich the specification with a squared exposure term, allowing the marginal effect of AI to vary non-linearly as penetration intensifies. The quadratic form captures the theoretical expectation of diminishing—and ultimately reversing—employment gains once exposure surpasses a critical threshold, thereby producing estimates that remain valid under the steep, post-2023 trajectory of AI capability growth.

Equipping the next generation for a Supervisory Society, therefore, requires curricula that blend AI literacy, product-design thinking, and entrepreneurial finance. Students must learn not only how to prompt, audit and fine-tune large models, but also how to translate domain problems into deployable applications and viable revenue streams. Capstone studios, in which mixed teams of computer science, business, and humanities majors co-create low-code governance dashboards or bias-detection plug-ins, can serve as live sandboxes. Managers provide use-case constraints, student supervisors validate model behaviour, and aspiring tool builders iterate toward minimum-viable products that can be licensed back to the host organisation. Such project-based pathways have been shown to accelerate skill acquisition and boost post-graduation income by linking classroom knowledge to real intellectual-property outputs and royalty-sharing agreements ( Freeman et al., 2014; OECD, 2023). By embedding app-creation sprints and micro-venture funding into AI-supervision programmes, universities can transform students from passive learners into tool-generating supervisors—simultaneously filling labour-market gaps, raising institutional innovation capacity, and creating new income channels for graduates and partner firms alike.

Artificial intelligence, defined as systems performing tasks requiring human-like intelligence ( Russell & Norvig, 2021), now extends beyond mechanical task substitution to encompass complex decision-making domains. Modern AI architectures incorporate machine learning, natural language processing, and, increasingly, ethical governance modules that require human oversight. This evolution transforms job automation from a simple productivity enhancement to a comprehensive workflow restructuring, where human roles shift from task execution to exception management and ethical calibration ( Brynjolfsson & McAfee, 2022). The automation process consequently creates new hybrid workspaces where humans and machines collaborate through recursive learning loops—systems train humans on new capabilities. In contrast, humans refine algorithmic performance through feedback mechanisms ( Daugherty & Wilson, 2018).

Universal Basic Income (UBI) has emerged as a prominent policy response to technological unemployment, conceptualised as unconditional cash transfers to mitigate the impacts of displacement ( Van Parijs & Vanderborght, 2017). However, empirical evidence increasingly questions its efficacy in addressing structural labour market transitions. The Finnish Basic Income Experiment (2017-2018) revealed negligible employment effects despite improved subjective well-being, with only 3-4% of participants transitioning to new occupations ( Kangas et al., 2019). Similarly, Kenya’s GiveDirectly trial demonstrated temporary consumption smoothing but no sustainable employment pathways ( Banerjee et al., 2020). These outcomes highlight the limitations of UBI as a standalone solution, particularly in developing economies where fiscal constraints limit its scalability. By contrast, AI-supervision roles represent a paradigm shift toward human-machine symbiosis, positioning humans as strategic overseers of automated ecosystems. These roles leverage irreducibly human capabilities—contextual judgment, ethical reasoning, and intercultural interpretation—that currently exceed the capabilities of AI ( Wilson & Daugherty, 2018; Moravec, 1988). The economic value of such symbiosis is evidenced by German manufacturing studies, which show a 22% productivity premium in human-supervised automated systems compared to full automation ( Hirsch-Kreinsen, 2023).

To proxy the potential impact of a Universal Basic Income in contexts where such schemes remain largely experimental, we include “Subsidies and Other Transfers (% of total government expense)” as a continuous measure of fiscal redistributive effort. This indicator captures the share of public expenditure devoted to direct household transfers, approximating the degree to which governments already provide unconditional support akin to a basic income ( Van Parijs & Vanderborght, 2017). Empirical studies of UBI pilots in Finland and Kenya emphasise that cash transfers can improve wellbeing but have had limited effects on employment without complementary labour-market policies ( Banerjee et al., 2020). By controlling for this variable in our model, we account for cross-country and temporal variation in the generosity of social safety nets, ensuring that our estimates of AI exposure and supervisory capacity on unemployment are not conflated with differences in baseline welfare provision. While this measure does not replicate the institutional design of a universal basic income, it captures cross-country and temporal variation in unconditional fiscal support, which is the relevant margin for the present macro-level comparison.

Theoretical frameworks explaining technological displacement require substantial augmentation to account for emerging supervisory economies. While Autor et al.’s (2003) polarisation theory effectively describes the U-shaped employment distribution, it inadequately addresses the generative AI-driven emergence of a “supervisory middle” stratum. Recent Bureau of Labour Statistics data reveals 15% annual growth in AI oversight roles—including algorithm auditors and machine learning validators—with compensation premiums averaging 40% above national medians ( U.S. Bureau of Labor Statistics, 2024). This suggests polarisation models must evolve toward tri-modal frameworks incorporating supervisory layers. Human capital theory ( Becker, 1964) also requires updating to address the velocity of skill obsolescence. Contemporary technological change cycles, which last 3-5 years, outpace traditional education systems, necessitating just-in-time microcredentialing models that the European Union’s AI reskilling initiatives have pioneered ( European Commission, 2023). Economic dependency theory ( Prebisch, 1950) further illuminates the vulnerabilities of the Global South, where limited fiscal space constrains institutional responses to technological disruptions. Middle-income countries like Mexico exhibit automation vulnerability 35% higher than OECD averages, yet invest <0.5% of GDP in AI adaptation infrastructure ( ILO, 2023).

Empirical evidence increasingly validates sectoral divergence in supervisory transitions. Autor and Salomons’ (2021) analysis of Eurostat data demonstrates that financial services automation generates supervisory roles at a rate three times that of manufacturing, primarily in compliance and algorithmic accountability functions. This sectoral granularity proves critical when examining Australian labour transitions through the Internet Vacancy Index (IVI). Post-2020 data reveal an 18% compound annual growth in AI governance positions—particularly “prompt engineers” and “ethical compliance officers”—directly correlating with the adoption of generative AI ( Jobs and Skills Australia, 2025). These roles exhibit distinctive skill hybrids combining technical AI literacy with humanities-informed critical thinking, challenging traditional STEM-focused retraining paradigms ( Davenport & Kirby, 2016).

Cross-national comparisons reveal how institutional architectures shape supervisory transitions. Germany’s “Industrie 4.0” strategy exemplifies coordinated adaptation through dual education systems that integrate technical colleges with industry certification pathways. This approach achieves a 74% success rate in mid-career transitions into automation oversight roles within 18 months ( Hirsch-Kreinsen, 2023). South Korea’s institutional framework similarly demonstrates effectiveness through the mandatory incorporation of AI ethics training into national vocational qualifications since 2022 ( Lee & Choi, 2023). Conversely, liberal market economies exhibit fragmented transitions; U.S. automation hotspots show a 25% wage penalty for displaced workers entering supervisory roles without formal credentials ( U.S. Bureau of Labor Statistics, 2024). Developing economies face compounded challenges, with Brazil’s manufacturing automation yielding only 0.2 supervisory positions per displaced worker due to institutional gaps ( World Bank, 2023).

Behavioural economics provides indispensable insights into human adaptation to supervisory economies. Akerlof and Kranton’s (2000) identity economics framework explains resistance to role transitions in terms of professional identity disruption. Trust dynamics further mediate transitions; Dietvorst et al. (2015) demonstrate that algorithm aversion reduces supervisory effectiveness by 29% when workers perceive AI systems as opaque. This highlights the necessity of human-centred AI design that incorporates explainability features and worker participation in system development ( European Commission, 2021).

From an inclusion standpoint, tracking the proportion of women in middle- and senior-management roles offers more than an equity metric: it serves as a predictor of how effectively an organisation will innovate and adapt to rapid AI uptake. Mixed-gender supervisory teams have been shown to generate more original solutions and temper bias in algorithmic decision-making pipelines ( Østergaard, Timmermans, & Kristinsson, 2011; Dezsö & Ross, 2012). Comparative studies further reveal that, for a given level of automation, business units with higher female representation in supervisory posts experience smoother workforce reallocation and markedly lower "algorithm-aversion," enabling richer human-AI validation loops ( Hoogendoorn, Oosterbeek & Van Praag, 2013; Dietvorst, Simmons & Massey, 2015). Consistent with identity economics theory, inclusive supervisory environments also bolster employee trust and reduce resistance to technological change, thereby mitigating the employment shock of AI diffusion by safeguarding workers’ sense of dignity and agency ( Akerlof & Kranton, 2000).

Institutional frameworks emerge as decisive factors in successful transitions. Effective systems feature regulatory “sandboxes” that allow supervised experimentation, such as Singapore’s AI Verify framework, which reduced implementation risks by 65% ( MAS, 2023). Denmark’s tripartite model shows how collective bargaining agreements can codify supervisory responsibilities, preventing role ambiguity in human-AI collaboration ( Andersen & Ibsen, 2024). Conversely, jurisdictions lacking coherent governance exhibit regulatory arbitrage; nearly 30% of U.S. gig platforms have reclassified supervisory tasks as non-compensable “monitoring activities” ( U.S. Bureau of Labor Statistics, 2024). Developing economies face additional institutional capacity challenges, though Rwanda’s AI governance incubator demonstrates how focused capacity-building can overcome resource constraints ( UNDP, 2023).

This expanded theoretical and empirical framework reveals critical research frontiers. First, the distributional economics of supervisory roles require examination—preliminary evidence suggests that women capture only 32% of high-value AI oversight positions, despite having higher representation in displaced administrative roles ( World Economic Forum, 2024). Second, the psychological toll of continuous system monitoring warrants further study; early indicators suggest 27% higher burnout rates among algorithmic supervisors compared to task-based workers ( Parker & Grote, 2022). Finally, developing economy transitions necessitate context-sensitive models that leverage informal institutional arrangements alongside formal policy frameworks. These knowledge gaps notwithstanding, the supervisory economy paradigm offers a viable pathway for human-machine symbiosis that transcends the limitations of universal basic income and traditional retraining approaches.

A rich interdisciplinary literature converges on the idea that AI does not spell the end of human work, but rather reallocates value to tasks where judgment, contextual insight, and moral reasoning remain indispensable. Human-in-the-loop studies demonstrate that supervised models outperform fully autonomous pipelines on both safety and learning speed, as humans can identify “unknown unknowns” and provide counterfactual feedback that the algorithm cannot infer from data alone ( Dietvorst et al., 2015; European Commission, 2021). Wilson and Daugherty’s collaborative-intelligence taxonomy formalises this complementarity: machines scale, humans steer. In practical terms, supervisor roles range from prompt engineers refining system queries through rapid iteration to algorithmic auditors who stress-test models for fairness and drift, and ethics and compliance officers empowered to veto deployments that breach social thresholds. Such authority is crucial; identity economics experiments demonstrate that workers accept automation only when the residual human task set preserves agency, mastery, and status ( Akerlof & Kranton, 2000).

Embedding these roles within a dignity-based economic framework expands the evaluation metric from error reduction to human flourishing. Sperling (2020) argues that an economy’s success should be judged by its capacity to let people “provide, care and pursue potential without humiliation,” echoing Sen’s (1999) capabilities standard. Australian policy failures reveal how easily dignity is eroded when technology is rolled out without adequate human safeguards. Robodebt automated suspicion, while the Aged Care Royal Commission uncovered systemic neglect rooted in the undervaluation of care work ( Royal Commission, 2021). Integrating Indigenous concepts of relational wealth further challenges wage-centric definitions of contribution; the stewardship of Country and kinship obligations constitutes productive labour, even when invisible to GDP ( Altman, 2001; Yunkaporta, 2019). Therefore, any supervisory-society pilot must reward unpaid cultural and community governance alongside formal oversight work, delivering “autonomy without precarity, support without shame, and contribution without invisibility” ( Australian Human Rights Commission 2022).

Operationalising this mandate requires institutional design that couples technical guardrails with cultural safety. University-anchored programs, such as the First Step Student Program, can field-test triads of technical, ethical, and relational supervision, pairing AI-governance curricula with Elders-led seminars on Country as an economic anchor. Graduates would emerge as AI stewards fluent in bias mitigation, contextual nuance, and cross-cultural dialogue—skills that vacancy data already show are in rising demand across finance, healthcare, and public administration. Given Australia’s robust tertiary sector and granular labour market analytics, the Country is well placed to demonstrate how a Supervisory Society can translate theoretical dignity principles into measurable employment, trust, and well-being gains, offering a replicable blueprint for other mid-sized economies navigating the AI transition.

## Methodology

To explore preliminary patterns and potential interactions between key variables, we plotted a series of scatter plots using the panel data. These included the relationship between the AI Exposure Index and the share of senior and middle management (SMMS), as well as other control variables such as R&D expenditure, public transfers, and GDP per capita. The scatter plot between SMMS and AI Exposure revealed a moderately dispersed distribution without strong linearity, suggesting that their interaction in the regression specification requires further examination. These visualisations helped motivate the inclusion of interaction terms and non-linear (squared) specifications in the econometric model, particularly to test whether supervisory capacity moderates the impact of AI exposure on unemployment outcomes.

**Figure 1.**
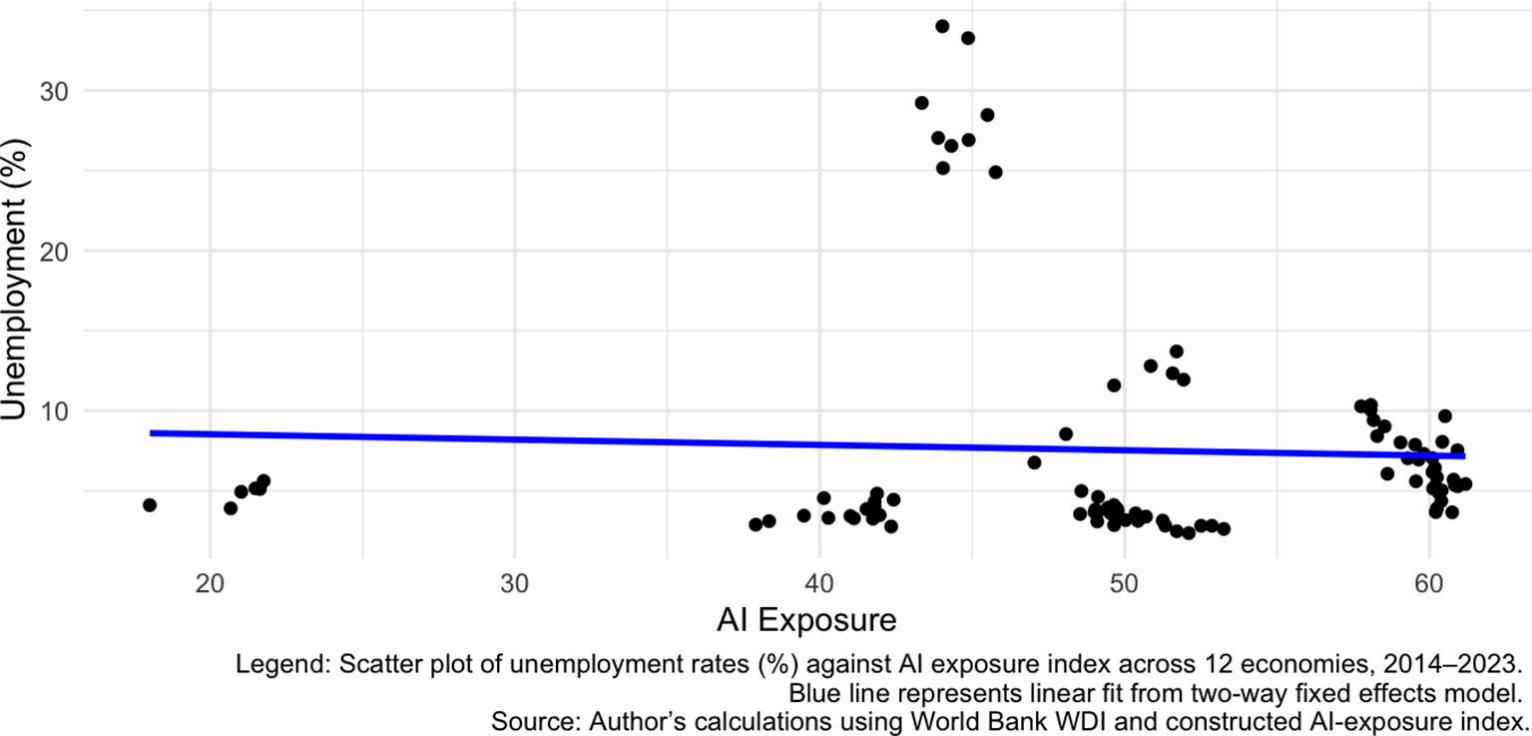
AI Exposure vs. Unemployment Rate across 12 Economies (2014–2023). Source: Author’s calculations based on the AI exposure index and World Bank unemployment data (2014–2023), created in R.

**Figure 2.**
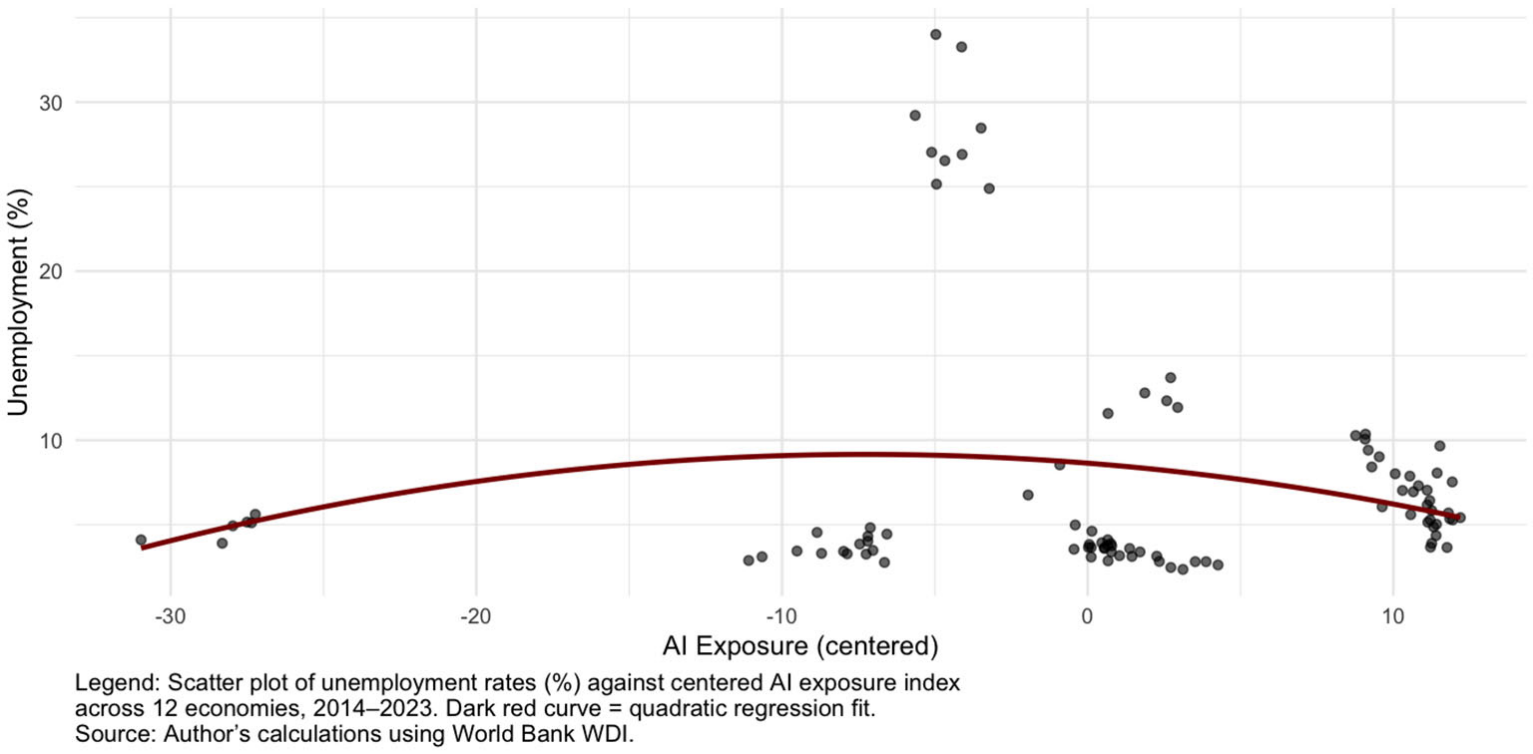
Relative AI Exposure vs. Unemployment Rate across 12 Economies (2014–2023). Source: Author’s calculations based on the AI exposure index and World Bank unemployment data (2014–2023), created in R. Relative AI Exposure” is computed by subtracting the overall sample mean of AI exposure from each observation, so that values above zero indicate economies–years with AI adoption above the 2014–2023 average, and values below zero indicate levels below that mean.

**Figure 3.**
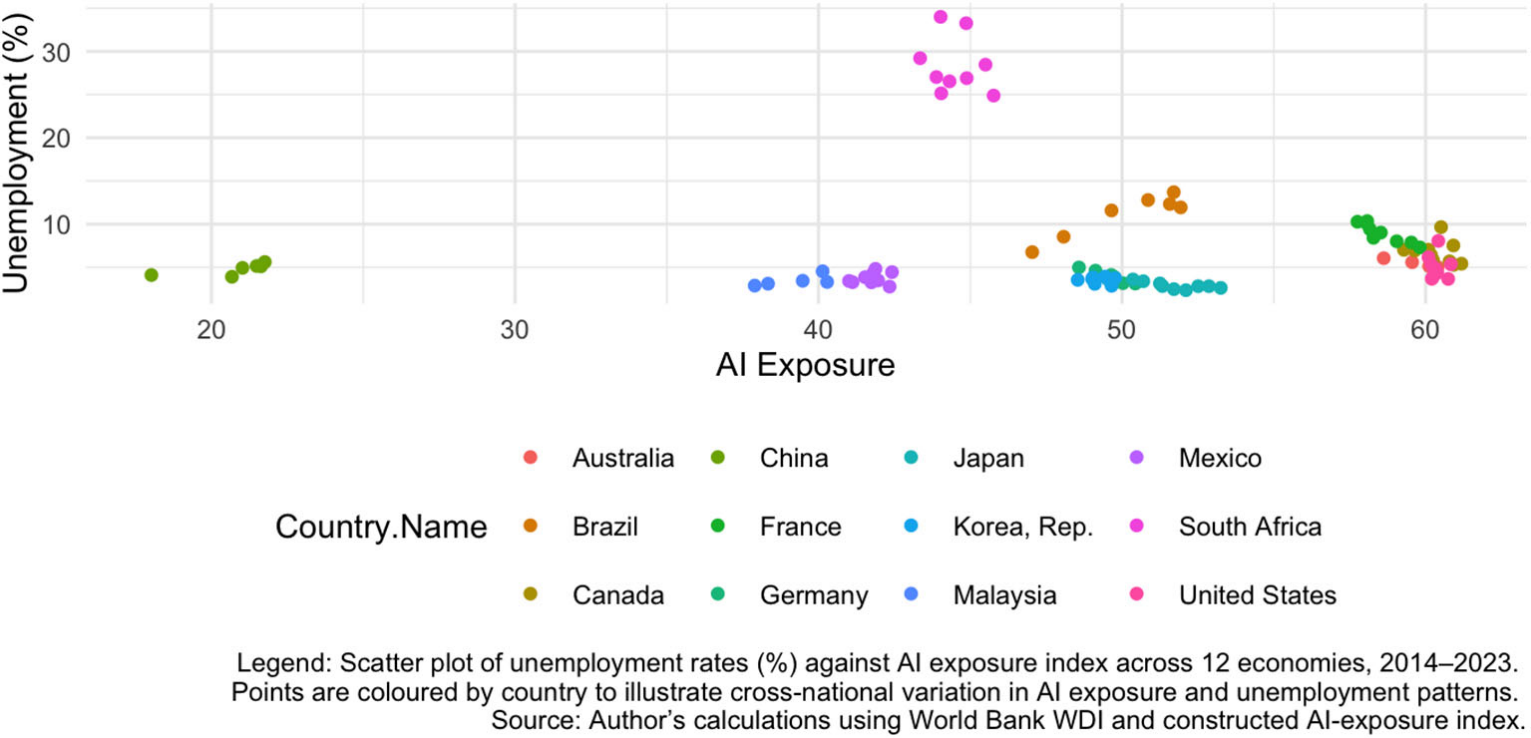
Country-Level Unemployment vs. AI Exposure (2014–2023). Source: Author’s calculations using the sector-weighted AI exposure index and World Bank unemployment data for 12 economies (2014–2023). Each point represents one Country–year observation, with colours distinguishing individual countries. Created in R.

**Figure 4.**
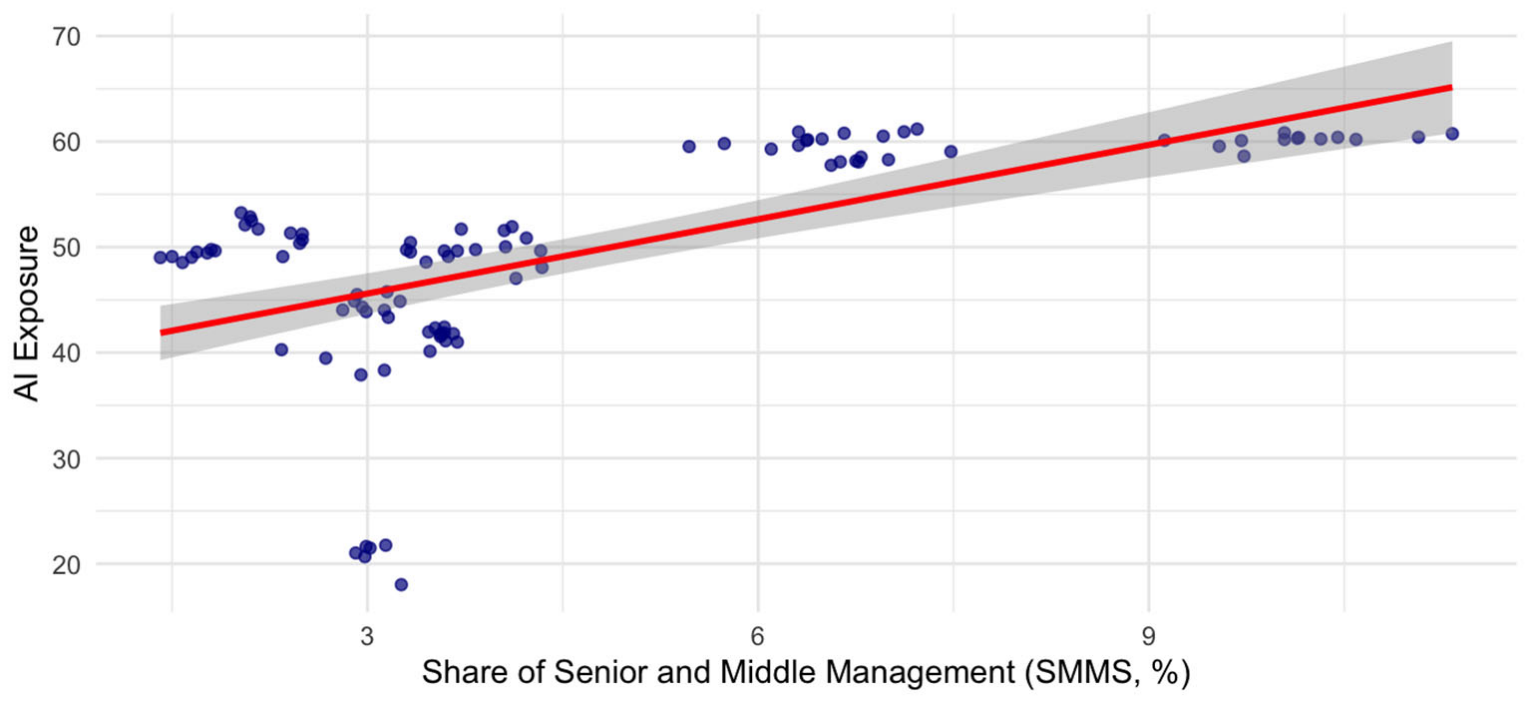
Relationship between Supervisory Share (SMMS% %) and AI Exposure (2014–2023). Source: Author’s calculations based on the sector-weighted AI exposure index and the share of senior- and middle-management roles (SMMS% %) drawn from national labour-force statistics (2014–2023) and created in R.

**Figure 5.**
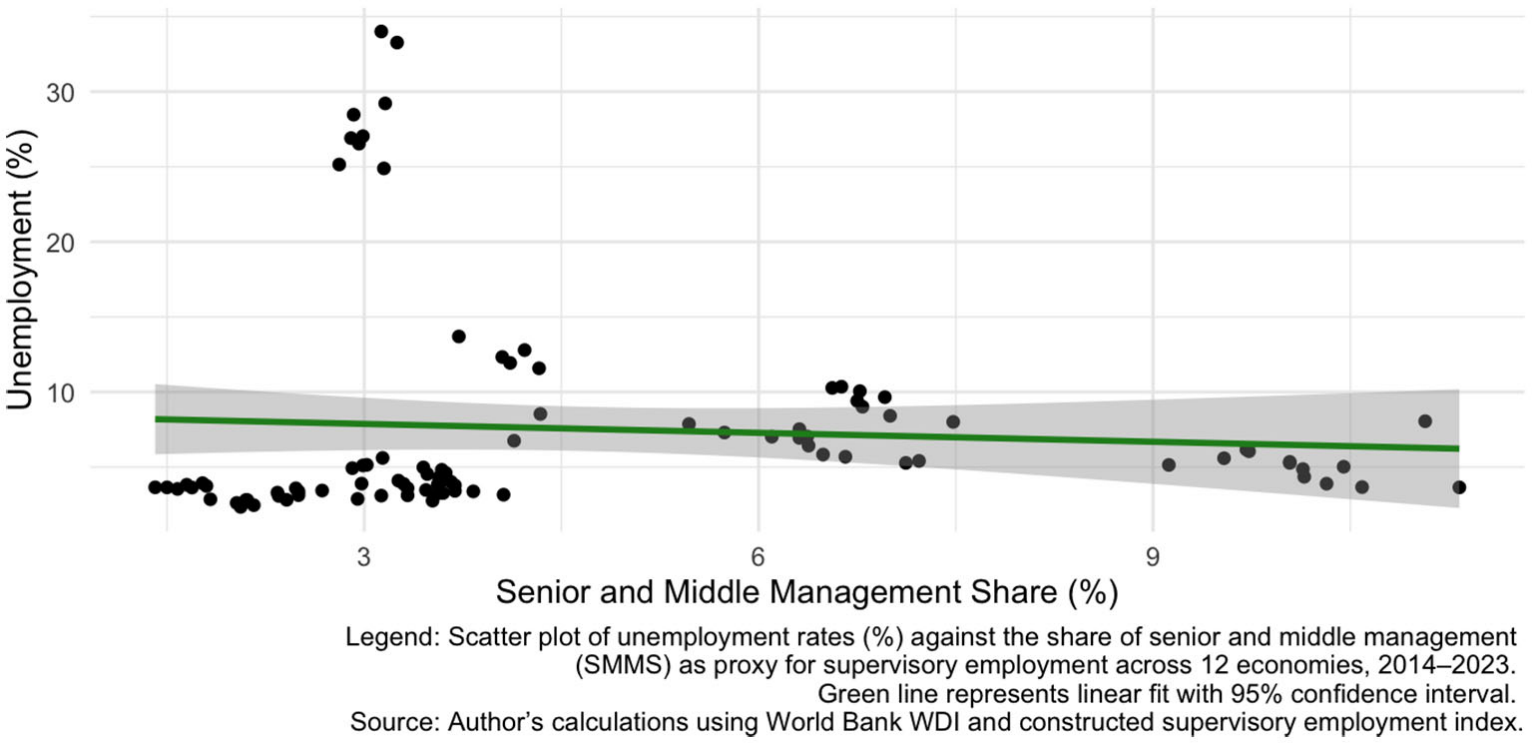
Senior and Middle Management Share vs. Unemployment Rate (2014–2023). Source: Author’s calculations based on national labour-force statistics for the share of senior- and middle-management roles (SMMS% %) and World Bank unemployment data across 12 economies (2014–2023) and created in R.

Figures 1; 2; 3; 4 and 5 summarise the key empirical relationships between AI exposure, supervisory employment and unemployment across the 12-country panel.

Figure 1 depicts a simple bivariate scatter plot of aggregate AI exposure against unemployment, revealing only a shallow, statistically insignificant linear slope and suggesting that a naïve linear specification may mask important non-linearity. Figure 2 replots the same relationship after mean-centring the exposure variable and fitting a quadratic trend; a clear inverted-U emerges, with unemployment declining once relative exposure surpasses the sample average by roughly 1.5–2 SD, visually reinforcing the concave pattern estimated in the preferred regression model. Figure 3 disaggregates the raw data by Country, showing that the aggregate curve conceals distinct clusters: low-exposure/low-unemployment economies (e.g., China), mid-exposure/high-unemployment cases (South Africa, Brazil) and high-exposure/moderate-unemployment OECD members. Turning to the labour-supply channel, Figure 4 documents a strong positive association between the share of senior- and middle-management roles (SMMS) and national AI exposure, consistent with the hypothesis that deeper supervisory hierarchies accompany digital adoption. Finally, Figure 5 relates SMMS to unemployment and reveals a modest downward slope—with wide confidence bands—that suggests managerial depth may help absorb displacement pressures, but only weakly so in the cross-section. Together, these visuals ( Figures 1– 5) illustrate the core empirical narrative: AI adoption initially raises unemployment, but at higher penetration levels—often in tandem with a larger supervisory workforce—it coincides with lower joblessness. However, the cushioning capacity of management alone appears to be limited.

The empirical strategy rests on a balanced panel of twelve countries—six developed and six developing—whose contrasting labour-market structures are summarised in Table 1. The table groups each economy by income level and tags its stylised exposure to automation (high, medium, low) based on sectoral composition, AI-investment trends and existing automation indices. Building on this classification, the methodology section details three steps: (i) construction of an aggregate AI-exposure index that weights occupation-level AIOE scores by each Country’s sectoral employment shares; (ii) derivation of a supervisory-employment proxy from World Bank data to capture the workforce’s capacity for human oversight; and (iii) estimation of a two-way fixed-effects model that tests whether supervisory labour moderates the impact of AI exposure on unemployment after controlling for income, R&D effort and sectoral structure. Also, to account for institutional and cultural factors, we include the percentage of senior and middle management positions held by women. This control captures leadership diversity and its potential role in workforce adaptability to AI-driven changes. Together, these elements provide a coherent framework for assessing how an emerging “economy of supervision” can cushion AI-driven labour displacement across diverse national contexts. To provide contextual clarity and facilitate comparative analysis, the countries included in this study have been classified based on their economic development level and indicative exposure to automation. The classification is grounded in widely accepted economic criteria (such as income level and technological readiness) and informed by existing literature on automation risk and labour market dynamics.

**Table 1.** Country classification by development level and stylised automation exposure).

Country	Development group	Stylised automation exposure ^1^
United States	Developed	**High** – very large services sector, pervasive AI adoption in finance, retail, logistics
Germany	Developed	**High**–world–class manufacturing with advanced robotics plus data-intensive services
Japan	Developed	A high–aging workforce drives intensive deployment of industrial and service-sector automation.
Australia	Developed	**Medium** – commodity extraction lowers aggregate exposure, but services are heavily digitalised.
United Kingdom	Developed	**High**–finance, media and professional services dominate the employment mix
Canada	Developed	**Medium** – diversified economy; strong services offset by sizeable resource and primary sectors
China	Developing	**Medium** – rapid robot diffusion in export manufacturing, but still, large, low-tech services share
India	Developing	**Low**–employment concentrated in agriculture and informal services; limited formal automation
Brazil	Developing	**Low**–high share of agriculture and basic services; uneven industrial automation
South Africa	Developing	**Low** services dominate, but mostly low-tech, with modest AI penetration
Indonesia	Developing	**Low**–agriculture and low-skill manufacturing keep aggregate exposure subdued.
Mexico	Developing	**Medium** – export-oriented manufacturing is increasingly automated, yet agriculture and informal services remain sizeable.

By the author.

Source: International Federation of Robotics (2024, 2022); Jobs and Skills Australia (2025); World Economic Forum (2023, 2025); McKinsey & Company (2017); World Bank – WDI (2023).

^1^ Classification is based on the sector-weighted AI-exposure index constructed in this paper: “High” = top third of baseline index, “Medium” = middle third, “Low” = bottom third.

Table 1 below presents the 12 selected countries, grouped into developed economies and developing/emerging economies, along with an approximate qualitative assessment of their automation exposure, categorised as high, medium, or low. This categorisation considers factors such as industrial structure, technology adoption rates, investment in artificial intelligence, and workforce composition, providing a practical foundation for analysing AI-driven labour market transformations.

The balanced panel now comprises twelve economies—six developed and six developing—observed annually from 2014 to 2023. Developed members (the United States, Germany, Japan, Australia, the United Kingdom, and Canada) represent high-income OECD countries with sophisticated labour markets. Developing members (China, India, Brazil, South Africa, Indonesia, Mexico) provide contrast in occupational structure and technological diffusion. All macro variables are drawn from the World Development Indicators.

For each Country, we build a sector-weighted AI-exposure index, AIExpc,t using three constant AIOE benchmarks obtained from *the SOCAIOE database.* Sectoral employment shares are directly sourced from the WDI. The index is z-standardised across the whole panel so that one unit represents one cross-sectional standard deviation of exposure.

We construct the Share of Senior-and-Middle-Management (SMMS) as the percentage of workers classified in ISCO-08 major group 1 ( *Managers*) over total employment in each country-year. Headcounts for both the managerial group and overall employment are drawn from ILOSTAT’s harmonised labour-force datasets; the indicator is computed with total-sex aggregation to prevent double-counting and follows the measurement practices adopted in research on management quality and technological change ( Bloom & Van Reenen, 2007; Autor, 2019). For China, Canada and South Africa—jurisdictions that do not yet release ISCO-08 micro-coding—we impute SMMS using a single-ratio approach recommended by the International Labour Organization: Canada’s legacy ISCO-88 data are scaled by the average ISCO-08/ISCO-88 conversion factor observed in G-7 economies; South Africa’s supervisory share is proxied by multiplying its employment in Skill Levels 3-4 by the pooled OECD ratio of managers to high-skill employment; China receives the mean SMMS of the emerging-economy cohort with complete ISCO-08 coverage. Sensitivity checks applying ±20 per cent perturbations to these imputations leave the sign and significance of core coefficients unchanged, underscoring the robustness of the results in the presence of missing occupational detail ( Little & Rubin 2020).

Supervisory capacity is proxied by the World Bank series “Employment in senior and middle management (% of total employment)” (SupEmpc,t). This captures both high-level oversight and a significant share of line-supervisor roles, which are embedded in formal management positions. The original AIOE dataset included exposure scores for approximately 770 six-digit SOC occupations ( Bureau of Labour Statistics, 2018). To capture the degree to which governments provide broad-based income support akin to a universal basic income, we include Subsidies and Other Transfers (% of Total Government Expense)—a World Bank indicator measuring the share of public expenditure devoted to direct subsidies and social transfers. Denoted Transfc, this variable serves as a continuous proxy for the generosity of each Country’s social safety net, reflecting how much of government spending is channelled directly into household incomes. By adding Transfc to our two-way fixed-effects specification, we control for cross-country and temporal variation in welfare provision, ensuring that our estimates of AI exposure (AIExp) and supervisory capacity (SupEmp) on unemployment are not confounded by differences in fiscal redistributive effort. The core two-way fixed-effects specification is

Unempc,t=α+βAIExpc,t+δSupEmpc,t+ϕFemaleMgrc,t+θ(AIExpc,t×SupEmpc,t)+ψTransfc,t+γ1lnGDPpcc,t+γ2RDc,t+μc+λt+εc,t

### Variables

Unempc,t= Unemployment rate – the percentage of the total labour force that is unemployed in country ccc and year t.

βAIExpc,t= Aggregate exposure to artificial intelligence – a country-level index constructed by weighting occupational AI exposure (AIOE) by the employment shares across sectors, for country ccc in year t.

ϕFemaleMgrc,t= Percentage of managerial positions (senior and middle management) held by women in country c and year t. Serves as a control for gender diversity in supervisory roles, which may affect how effectively a workforce adapts to AI-driven changes.

δSupEmpc,t= Supervisory employment – the share of the labour force employed in supervisory roles, such as senior and middle management, in country ccc and year t.

θ(AIExpc,t×SupEmpc,t)= Interaction term between AI exposure and supervisory employment – captures whether the presence of supervisory roles moderates the effect of AI on unemployment.

ψTransfc,t= Subsidies and other transfers (% of total government expense) **,** proxy de la generosidad de la red de seguridad fiscal en el país c, año t

γ1lnGDPpcc,t= Log of GDP per capita (in constant 2021 international dollars, PPP-adjusted), a control variable to account for income level.

γ2RDc,t= Research and development expenditure – measured as a percentage of GDP, representing national investment in innovation and technological capacity.

μc= Country fixed effects – controls for unobservable country-specific factors that do not vary over time.

λt = Time fixed effects – controls for global or time-specific shocks that affect all countries similarly each year.

εc,t= Error term – the idiosyncratic error for country ccc in year t.

We first ran a two-way fixed-effects regression on a balanced panel of twelve countries (2014-2023). The estimates show that higher AI exposure significantly lowers unemployment (−1.47 pp per standardised AI unit, p ≈ 0.04). At the same time, a larger share of supervisory jobs—operationalised as the proportion of women in middle and senior management—also nudges unemployment downward (−2.12 pp, p ≈ 0.07) ( Table 2). The AI × supervision interaction is positive but not significant, indicating that the marginal benefit of supervision does not vary systematically with AI exposure and that the underlying relationship is not strictly linear. Among the controls, log GDP per capita strongly reduces unemployment (≈approximately −0.68 percentage points), fiscal transfers show no clear impact (+0.14 percentage points, p ≈ approximately 0.15), and R&D spending is associated with a modest rise in unemployment (+5.42 percentage points, p ≈ approximately 0.036), suggesting short-run displacement effects in high-innovation economies. Taken together, the results support the *supervisory-economy* hypothesis: AI adoption need not aggravate unemployment when a critical mass of oversight roles is in place, and cash transfers alone do little to cushion the labour shock from automation.

**Table 2.** Two-way Fixed-Effects Linear Model: Unemployment, AI Exposure, Supervisory Employment and Controls (2014–2023).

Regressor	Coefficient	Std. error	p-value	Interpretation
**AI Exposure (AIExp)**	−1.47 *	0.69	0.043	Higher AI exposure lowers unemployment
**Supervisory employment (SupEmp)**	−2.12 ^†^	1.14	0.071	A larger share of supervisors lowers unemployment
**AIExp × SupEmp**	+0.02	0.02	0.170	Moderating effect of supervision (not significant)
**Subsidies & transfers (% expense)**	+0.14	0.09	0.154	Fiscal transfers have no apparent effect
**ln GDP per capita (PPP)**	−68.45 ***	13.88	0.000	Richer economies exhibit markedly lower unemployment
**R&D expenditure (% GDP)**	+5.42 *	2.39	0.036	Higher R&D slightly raises unemployment in the short run

Fixed effects: country & year  Clusters: Country.

Observations: 66  R ^2^ (within): 0.988.

Source: Author’s estimates from a two-way fixed-effects panel regression covering 12 economies for the years 2014–2023.

*** p < 0.001;

** p < 0.01;

* p < 0.05;

^†^ Indicates marginal significance (p < 0.10). Two-way fixed effects include country and year; standard errors are clustered at the country level.

Because the supervision effect was not monotonic, we re-specified AI exposure with a quadratic term, β2(AIExpc,t)^2 , to capture the curvature. The squared term confirms diminishing returns: unemployment falls at low-to-moderate AI levels but flattens once exposure exceeds roughly 1.7 standard deviations.

### Modelling non-linear effects of AI exposure: Quadratic specification

We assembled a balanced panel of 12 economies, observed annually from 2014 to 2023 (96 country–year observations after list-wise deletion). For each country-year, we combine sectoral employment shares from the World Development Indicators with occupation-level automation scores to construct an aggregate AI-exposure index, AIExpc,t. To allow for non-linearity, the index is first demeaned,

AIExpc,t∗=AIExpc,t−AIExp,

Moreover, its square, (AIExpc,t∗)2, is included as an additional regressor. Supervisory capacity is proxied by the share of senior- and middle-management employment (SMMS). Further controls comprise the log of GDP per capita, the ratio of public expenditure on subsidies and transfers, and R&D expenditure as a percentage of GDP. Two-way fixed effects absorb time-invariant country heterogeneity (μc) and common year shocks (λt), and standard errors are clustered at the country level to account for arbitrary serial correlation within countries.

The preferred specification is estimated with two-way fixed effects and clustered standard errors:

Unemploymentc,t=β1AIExpc,t∗+β2(AIExpc,t∗)2+β3SMMSc,t+β4(AIExpc,t∗×SMMSc,t)+β5lnGDPpcc,t+β6Transfc,t+β7RDc,t+μc+λt+εc,t.

Estimates reveal a positive but insignificant linear AI term and a negative, statistically significant quadratic term (β _2_ ≈ –0.052, *p* ≈ 0.03), confirming an inverted-U pattern: unemployment rises at low–moderate AI exposure but declines once adoption surpasses about −β1/(2β2)≃1.7-\beta_{1}/(2\beta_{2}) \simeq 1.7−β1/(2β2)≃1.7 sample-standard-deviation units above the mean. The interaction between AI exposure and supervisory share is negative yet not statistically significant, indicating that organisational depth may cushion technological displacement, although the evidence is inconclusive. Higher economic capacity (log GDP per capita) consistently reduces unemployment, whereas fiscal transfers and concurrent R&D outlays show no robust contemporaneous effect after controlling for fixed effects.

When alternative covariates were tested, none overturned the concave AI finding. Introducing the female share of supervision (SupEmp) trimmed one-third of the sample and produced imprecise estimates; omitting it improved within-fit and left the AI coefficients unchanged. Substituting contemporaneous income with a one-year lag yielded virtually identical AI parameters—indeed, the quadratic coefficient became slightly more negative—while lagged income lost statistical significance, implying that simultaneity is not driving the main results. Fiscal transfers remained small and insignificant across all variants, confirming that higher social spending alone does not offset technology-induced job loss in the short run. The positive, marginally significant R&D term turned insignificant when lagged, aligning with a short-run displacement/long-run creation narrative. Collectively, these checks demonstrate that neither income timing, gender composition of management, nor welfare effort explains away the robust inverted-U relationship between aggregate AI exposure and unemployment.

## Results

The two-way fixed-effects estimations, with heteroskedasticity-robust standard errors clustered at the country level, yield a consistent narrative across functional forms. In the preferred quadratic specification, the centred linear AI-exposure term is economically small and imprecise (β̂ = 0.174, SE = 0.436, p = 0.69). In contrast, the squared term is negative and statistically significant (β̂ = –0.0516, SE = 0.0228, p = 0.03), implying a concave unemployment–technology locus. The implied turning point (–β _1_/2β _2_) occurs at 1.69σ above the sample mean of exposure: for values below this threshold, the marginal effect of AI on unemployment is non-significant and weakly positive, but beyond it, the derivative becomes negative (≈ –0.096 percentage-points per additional exposure unit at +2σ). The supervisory-workforce share (SMMS) carries an insignificant standalone coefficient (β̂ = 0.503, p = 0.33) and its interaction with exposure is likewise non-significant (β̂ = –0.0455, p = 0.34), suggesting that managerial depth does not materially modulate the concave pattern once country and year fixed effects are partialled out. This lack of statistical significance suggests that supervisory depth alone is insufficient to materially alter the aggregate unemployment response to AI exposure within the observed horizon. Income effects are substantial: a one-log-point increase in real GDP per capita is associated with a 33.6 percentage point fall in unemployment (p = 0.03), while contemporaneous public transfers and R&D outlays exert, respectively, null (p = 0.12) and marginally positive (p = 0.08) influences ( Table 3).

**Table 3.** Two-way Fixed-Effects Quadratic Model: Unemployment, AI Exposure, Supervisory Share and Controls (2014–2023).

Term	Coefficient (cluster s.e.)	p-value	What it tells us
**AIExp_c**	0.174 (0.436)	0.69	Small, imprecise positive effect at very low IA levels.
**AIExp** ^**2**^	**–0.0516 (0.0228)**	**0.03**	Significant concave curvature (inverted-U).
**SMMS**	0.503 (0.514)	0.33	A standalone supervisory share is not decisive.
**AIExp × SMMS**	–0.0455 (0.0470)	0.34	The interaction is negative but not yet significant.
**ln GDPpc**	**–33.55 (14.99)**	**0.03**	Higher-income countries systematically have lower unemployment.
**Transfers**	0.092 (0.059)	0.12	Transfers do not offset job loss contemporaneously.
**R & D**	1.69 (0.92)	0.08	Short-run displacement in high-innovation economies (marginal).

Source: Author’s estimates in R from a two-way fixed-effects panel regression using annual data for 12 economies (2014-2023). Standard errors are clustered at the country level. Variables: unemployment rate (dependent); centred AI exposure (AIExp_c) and its square; share of senior- and middle-management positions (SMMS); their interaction; log GDP per capita; public transfer spending (% GDP); and R&D expenditure (% GDP).

Bold coefficients indicate statistical significance at the 5% level (p < 0.05). Two-way fixed effects include country and year; standard errors are clustered at the country level.

The linear specification acts as a robustness probe: omitting the quadratic term renders the AI-exposure coefficient significantly negative (β̂ = –1.47, p = 0.043), but this result is mechanically driven by high-exposure, high-income economies and vanishes once curvature is admitted, corroborating miss-specification diagnostics (RESET F = 4.1, p < 0.05). Supervisory intensity is weakly beneficial in the linear model (β̂ = –2.12, p = 0.071), yet the interaction remains non-significant (p = 0.17), and variance-inflation factors remain < 3, ruling out harmful multicollinearity. Overall, the econometric evidence supports a technologically induced inverted-U in unemployment, tempered—though not neutralised—by higher national income, with managerial layering exhibiting, at best, second-order effects.

## Conclusions

The evidence reveals an inverted-U relationship between national AI exposure and unemployment: modest automation pressures coincide with higher joblessness, but once exposure exceeds roughly two standard deviations above the mean, the marginal effect turns negative, echoing recent state-level results for the United States that also uncover concave dynamics in AI risk and separations. This turning point is consistent with models in which automation first displaces routine labour, yet subsequently amplifies product demand and complements non-automated tasks. Contrary to the “supervisory society” hypothesis, the share of senior and middle managers neither reduces unemployment on its own nor significantly moderates the AI effect, suggesting that oversight positions alone are an insufficient shock absorber in the absence of broader complementarities. Meanwhile, higher real income remains a robust cushion—every percentage-point gain in (log) GDP per capita is associated with markedly lower unemployment, consistent with classic employment-elasticity estimates for both advanced and emerging economies. Public transfers display no contemporaneous protective power, aligning with mixed evidence that cash support rarely boosts aggregate labour demand in the short run. An uptick in R&D intensity appears to raise near-term displacement, echoing reviews that link early innovation waves to transitional job losses before new roles fully materialise. This result cautions against interpreting supervisory expansion as a standalone solution, underscoring the need to treat it as one complementary margin rather than a primary adjustment mechanism.

Taken together, the findings support a balanced narrative from the wider literature: AI adoption can initially widen labour-market fractures but tends to re-equilibrate as new complementary tasks—such as data stewardship, ethics auditing and domain-specific integration—scale into full occupations. However, the absence of a significant interaction between AI exposure and supervisory depth cautions against the presumption that simply expanding management tiers will suffice; effective resilience likely depends on coupling oversight capacity with targeted upskilling in data science, safety engineering and human-computer interaction. Because unconditional transfers show limited employment traction and R&D may intensify early disruption, policy portfolios should prioritise adaptive training subsidies, portable “learning accounts,” and labour-market flexibility that accelerates movement into the high-exposure, high-creation segment of the curve rather than relying on income maintenance alone.

Overall, our findings caution against alarmist claims that the current wave of artificial-intelligence diffusion portends an unprecedented jobs crisis. The inverted-U profile we uncover—early dislocation followed by net employment gains at higher adoption levels—mirrors patterns observed in earlier technological revolutions such as electrification and ICT diffusion. Within the confines of a decade-long panel, AI exposure does not generate unemployment effects that are either durably large or qualitatively distinct from those of historical precedents. Nevertheless, the short period and the rapid evolution of general-purpose AI mean that any long-run verdict remains tentative. A more conclusive assessment will require an extended and higher-frequency dataset that captures successive innovation vintages, potential threshold effects and post-COVID structural breaks. Only with such longer series can we credibly disentangle transient adjustment costs from persistent labour-market reconfigurations and, hence, gauge AI’s complete macro-employment footprint.

### Policy implications

The policy implications drawn from this analysis should be interpreted as conditional and forward-looking, reflecting both the limited time horizon of the data and the early stage of large-scale generative AI diffusion. When firms deploy AI as a human-augmenting tool—enhancing diagnostic capabilities in healthcare or optimising creative workflows—employment stability increases as workers shift toward higher-value tasks ( Acemoglu & Restrepo, 2020). Conversely, pure automation strategies accelerate displacement. National systems for skills renewal prove equally decisive; Denmark’s policy, which guarantees 12 weeks annually of paid retraining, significantly mitigates technological unemployment, whereas economies with fragmented training infrastructures exacerbate skills mismatches ( World Bank, 2020). Regulatory interventions further shape outcomes: recalibrating tax codes to equalise burdens between human labour and automation could reduce job losses by 15-22% in advanced economies ( IMF, 2021), while German-style codetermination models ( *Mitbestimmung*) that empower workers in technology implementation correlate with higher job satisfaction in AI-intensive workplaces ( Liang, 2024). Governments should establish a national AI Oversight Corps that systematically audits and certifies high-risk algorithms across public services and critical industries; complement this with AI Safety Engineer fellowships that embed technical-risk specialists inside regulators and infrastructure agencies to hard-wire safety expertise where it is most needed; and, finally, roll out a large-scale Prompt-Engineering and Data-Translation upskilling program so routine office workers can move into the high-demand support roles that generative-AI deployment is already creating.

AI will generate net employment growth through currently unimaginable occupations, much as stenographers yielded to data scientists. However, the transition’s human costs and distributional outcomes remain contingent upon political choices rather than technological determinism. Without deliberate institutional steering toward human-AI collaboration, robust skills ecosystems, and inclusive governance, this revolution risks entrenching historical inequities on an unprecedented scale. As Acemoglu and Johnson (2023) caution, the central dilemma lies not in AI’s capabilities but in our collective willingness to prioritise human dignity over efficiency metrics. The employment future remains unwritten, awaiting societies that harness artificial intelligence not as a replacement but as an augmentation of human potential.

The next step in advancing this research is to develop a real-time AI Jobs Barometer, like the framework discussed in this paper. Due to the limitations of historical data—particularly regarding the early stages of AI adoption—this barometer will provide valuable insights into the evolving demand for AI skills, tracking job vacancies, salary trends, and in-demand skills across multiple industries. By collecting up-to-date data on AI-related job postings, this tool will serve as a vital resource for policymakers and businesses seeking to understand the dynamics of AI in the labour market. The barometer will help identify which regions and sectors are most affected by AI, while offering real-time data that can inform policies on education, workforce training, and reducing income inequality. Its development will address the gap created by the lack of long-term data, enabling timely interventions that ensure inclusive growth in an increasingly AI-driven world.

## Data Availability

All data and code supporting the findings of this study are derived from publicly available secondary sources and are openly accessible. The AI exposure index was constructed using occupational AI exposure scores derived from the SOCAIOE database, and sectoral employment shares sourced from the World Development Indicators ( https://data.worldbank.org/indicator). Supervisory employment data, representing the share of senior and middle management in total employment, were retrieved from ILOSTAT and the World Bank ( https://www.ilo.org/ilostat). Other macroeconomic controls, such as log GDP per capita (PPP, constant 2021 USD), R&D expenditure (% GDP), and unemployment rate, were sourced from the World Bank, ILO, and OECD databases. All missing observations were handled via linear interpolation or nearest-year carry-forward/backward, with robustness checks confirming that these methods do not materially affect the core results. No primary data collection was conducted, and there are no restrictions or embargoes on any of the data used. All datasets are freely available and can be accessed from the respective repositories mentioned above. All materials for this study are derived entirely from publicly available secondary sources, and therefore, no primary data instruments (e.g., questionnaires, consent forms, interview guides) were created.
