# The Concave Relationship Between AI Exposure and Unemployment: Reframing the Supervisory Economy as an Exploratory Moderation Test

**DOI:** 10.12688/f1000research.168512.3

**Published:** 2026-05-14

**Authors:** Peter Malliaros, W Alejandro Pacheco-Jaramillo

**Affiliations:** 1 Research, UrCommunity Ltda., Melbourne, VIC, 3051, Australia; 2 Faculty of Business, Government and Law, University of Canberra, Canberra, ACT, 2617, Australia

**Keywords:** Artificial intelligence (O33); Automation (O33); Supervisory economy (J21); Labour-market dynamics (J21); Unemployment (E24); Human capital (J24); Technological change (O33)

## Abstract

**Background:**

Rapid advances in general-purpose artificial intelligence are compressing automation timelines and renewing concern about technological unemployment. This article examines whether aggregate AI exposure is associated with unemployment in a cross-country panel, and whether a broad managerial-share proxy provides any evidence for the proposed “supervisory economy” mechanism.

**Methods:**

Using a balanced panel of 12 economies observed annually from 2014 to 2023, we construct a sector-weighted AI-exposure index and match it to labour-force data on unemployment, senior- and middle-management employment, public transfers, R&D, and GDP per capita. Two-way fixed-effects regressions are estimated linearly and with a quadratic AI term to test non-linearity within the observed support.

**Results:**

The preferred quadratic specification reveals an inverted-U association between aggregate AI exposure and unemployment: joblessness rises at low-to-moderate exposure but falls once exposure reaches the upper end of the sample distribution. The managerial-share proxy has no significant standalone effect and does not significantly moderate the AI-unemployment association.

**Conclusions:**

The most robust empirical contribution is the concave AI-unemployment relationship. The supervisory-economy argument should therefore be read as a conceptual and policy-research agenda rather than as a mechanism directly identified by the present proxy. Future work requires vacancy-level or occupation-level measures of AI governance, algorithmic-risk, model-monitoring and prompt-engineering roles to test the mechanism directly.

## Introduction

Artificial intelligence (AI) is no longer a distant prospect, but a general-purpose technology reshaping labour demand in real time. Seminal forecasts estimate that 40–50 per cent of current tasks in advanced economies are within the technical reach of automation, while successive World Economic Forum surveys predict that roughly one-quarter of all hours worked could be displaced by machines before 2030 ( Frey & Osborne, 2017; WEF, 2025). Empirical evidence already confirms the initial stages of this transition: in Europe and North America, mid-skill, routine-intensive occupations have contracted sharply, while employment has expanded in low-skill personal services and high-skill analytical roles ( Autor, 2015; Goos et al., 2014). A second, quieter trend is also visible in vacancy data: new roles are appearing whose core duties involve supervising, auditing or contextualising AI systems, including AI-governance analysts, algorithmic-risk managers and quality-assurance leads. The present article treats this supervisory-economy idea as an important conceptual proposition, but it does not claim to measure those AI-specific occupations directly in the cross-country panel.

Existing policy prescriptions struggle to accommodate this possibility. Mass-retraining initiatives are often outpaced by rapid model cycles, and universal basic income (UBI) pilots in Finland and Kenya have improved self-reported well-being without increasing sustained employment ( Kangas et al., 2019; Banerjee et al., 2020). What remains missing is a systematic evaluation of whether an economy can pivot from doing to supervising—and whether such a pivot outperforms cash transfers on both employment and fiscal grounds.

As breakthroughs in artificial intelligence now outpace the slow churn of conventional up-skilling systems, redirecting displaced workers into AI-supervisory roles emerges as a more realistic safeguard of economic dignity than relying on training programmes that inevitably lag the technological frontier.

This paper refines that gap. Anchored in employment-polarisation theory ( Autor, Levy & Murnane, 2003), human-capital theory ( Becker, 1964) and economic-dependency theory ( Prebisch, 1950), we use the supervisory economy as a theoretical lens for asking whether AI exposure follows a non-linear employment trajectory and whether general managerial depth is associated with greater labour-market resilience. The empirical test is deliberately narrower than the full concept: because comparable cross-country data on AI-governance, algorithmic-risk and model-monitoring vacancies are not yet available for the whole panel, senior- and middle-management share is used only as a coarse exploratory proxy for organisational oversight capacity. The revised hypotheses are therefore: (H1) AI exposure is non-linearly associated with unemployment; (H2) broad managerial intensity may moderate this association, but cannot by itself identify AI-specific oversight labour; and (H3) public transfers and R&D intensity may shape adjustment, without necessarily offsetting short-run displacement.

Methodologically, the study focuses on one cross-country empirical layer. A balanced panel of 12 OECD and partner economies observed annually from 2014 to 2023 is used to estimate the association between sector-weighted AI exposure and unemployment. The Australian vacancy examples and UBI evidence are used as contextual motivation and policy comparison, not as separately estimated components of the panel model.

The contribution is therefore more precise: the paper estimates a non-linear AI-unemployment relationship and reports an exploratory moderation test using broad managerial share. This design can inform governments about the macro-employment pattern associated with AI exposure, while also clarifying what remains untested: whether AI-specific oversight occupations expand quickly enough to absorb displaced workers.

## Literature review

Technological revolutions have consistently generated both profound anxiety and transformative economic progress throughout modern history. The canonical framework of *creative destruction*, as articulated by Schumpeter (1942), posits that technological innovation inherently dismantles established economic structures while simultaneously creating new industries and occupations. This dual process is evident in historical precedents: during Britain’s Industrial Revolution, agricultural employment plummeted from 80% to just 3% of the workforce, yet manufacturing absorbed displaced labourers through unprecedented productivity gains and the creation of new occupational categories ( Allen, 2009). Similarly, late 20th-century computerisation eradicated approximately 3.5 million routine clerical positions across OECD nations between 1980 and 2010 but concurrently generated 19 million new roles in cognitive services and non-automatable sectors ( Autor et al., 2003). These transitions validate economic theory’s long-term optimism regarding technological unemployment, as disruption is inevitable. However, compensation mechanisms have historically prevailed through demand expansion, occupational diversification, and productivity spillovers.

The contemporary artificial intelligence revolution, however, exhibits distinctive characteristics that challenge historical analogies. Unlike prior automation waves focused on manual or routine cognitive tasks, AI systems are increasingly demonstrating competence in domains that require advanced pattern recognition, contextual judgment, and creative synthesis—capabilities previously considered exclusively human ( Webb, 2020). Patent analysis reveals AI can currently perform 15% of tasks undertaken by top-quartile wage earners, including medical diagnostics, legal research, and algorithmic financial trading ( Webb, 2020). This cognitive breadth distinguishes AI from earlier technologies, threatening professional classes once insulated from automation. Moreover, the velocity of AI diffusion is unprecedented; generative tools like ChatGPT achieved 100 million users within two months, ten times faster than previous general-purpose technologies ( Our World in Data, 2023). Such acceleration compresses adaptation timelines for workers and institutions alike, potentially exacerbating displacement shocks.

Empirical evidence reveals divergent labour market impacts contingent on skill complementarity and institutional contexts. In OECD economies, occupations characterised by high computer usage exhibit 2.1% annual employment growth when integrated with AI, whereas low-digital-intensity occupations experience 1.7% reductions in working hours ( OECD, 2022). This bifurcation highlights AI’s dual role as both a productivity multiplier for digitally fluent workers and a substitute for others. Firm-level evidence from Taiwan indicates AI-adopting enterprises increased productivity by 14% while reducing non-AI hiring by 6.2%, suggesting task reorganisation rather than categorical job destruction ( Liang, 2024). Macroeconomic projections further support cautious optimism: the World Economic Forum (2020) anticipates 97 million AI-driven occupations emerging by 2025—primarily in machine learning, renewable energy, and care economies—offsetting 85 million displaced roles for a net global gain of 12 million jobs. Nevertheless, distributional consequences remain severe; workers lacking tertiary education face displacement probabilities three times higher than degree holders ( ILO, 2021).

The rapid advancement of artificial intelligence (AI) and automation technologies continues to fundamentally restructure global labour markets, generating profound concerns about employment sustainability and economic inequality. Frey and Osborne’s (2017) projection that 47% of U.S. employment faces a high automation risk has been substantiated cross-nationally through Organisation for Economic Co-operation and Development (OECD) studies, which show 14-32% job displacement probabilities across advanced economies ( Arntz et al., 2016). This technological disruption manifests as pronounced occupational polarisation, where employment growth concentrates at both skill extremes while mid-skill occupations experience disproportionate erosion ( Autor, 2015; Goos et al., 2014). Crucially, generative AI systems, such as large language models, have accelerated the displacement into cognitive domains previously considered resistant to automation. Recent analyses indicate that 80% of the U.S. workforce could see at least 10% of their tasks automated by generative AI, with roles involving writing, coding, and information processing facing 40-50% task exposure ( Eloundou et al., 2023; Felten et al., 2023). This represents a paradigm shift beyond traditional routineness-based displacement models, necessitating reconceptualisation of labour adaptation frameworks.

Profound distributional challenges nonetheless threaten to eclipse aggregate employment gains. Wage polarisation intensifies as AI-complementary workers command 17% premiums over those in substitutable roles ( Felten et al., 2019). Geographical disparities widen simultaneously; advanced economies exhibit 60% AI exposure, compared to 26% in low-income nations. However, developing regions face higher displacement risks due to occupational structures concentrated in automatable tasks and weaker social safety nets ( UNDP, 2023). Gender asymmetries compound these inequities, with women in OECD countries experiencing 9.6% job vulnerability compared to 3.5% among men—a disparity rooted in occupational segregation and digital access gaps ( ILO, 2021). These intersecting inequalities necessitate targeted policy innovations, including robot taxation schemes that fund portable reskilling accounts (modelled on Singapore’s SkillsFuture), strengthened collective bargaining frameworks, and ethical AI governance that prevents algorithmic discrimination.

Recent scholarship converges on the view that the tempo of artificial-intelligence breakthroughs now outstrips the cadence of formal up-skilling systems, creating a structural lag between the obsolescence of traditional tasks and workers’ ability to secure the competencies demanded by the digital economy. Frey and Osborne (2017) projected that nearly half of current jobs are technically automatable, yet vocational curricula remain locked into multi-year accreditation cycles. The gap has widened: laboratory estimates suggest that large language models such as GPT-4 can reconfigure up to 40 per cent of cognitive tasks within months of release ( Eloundou et al., 2023), leaving policymakers “running up a down escalator” ( Davenport & Kirby, 2016). Autor’s (2015) evidence shows that mid-skill jobs disappear faster than replacement roles can be generated, while human-capital theory implies that when the payoff horizon for new schooling shrinks, incentives to retrain erode ( Becker, 1964). Taken together, these dynamics reinforce this paper’s premise that pivoting displaced workers into AI-supervisory positions—where uniquely human judgement and ethical oversight remain indispensable—offers a more feasible absorption pathway than relying on up-skilling regimes that inevitably trail the technological frontier.

Despite extensive documentation of automation risks, significant knowledge gaps persist regarding sector-specific AI adoption trajectories and their precise relationship to the emergence of supervisory roles. Manufacturing automation, for instance, follows fundamentally different displacement patterns than service-sector AI implementation. Autor et al. (2022) demonstrate that healthcare and education automation generate 23% higher supervisory role demand than manufacturing, reflecting domain-specific requirements for human oversight in ethically sensitive contexts. This sectoral heterogeneity remains underexplored in current policy formulations, particularly concerning developing economies where labour market institutions face capacity constraints ( World Bank, 2023). Understanding these nuances is essential for designing targeted labour market interventions.

Because the dataset ends in 2023, precisely as generative AI diffusion accelerated, the analysis should be interpreted as evidence from the pre-generative-AI period and its earliest transition phase. This timing motivates a quadratic specification, since a constant-slope model is unlikely to capture adjustment dynamics across low, medium and high exposure. However, the estimates do not license a definitive post-2023 verdict. They identify a concave association within the observed 2014–2023 support and should be stress-tested as newer labour-market data become available.

The supervisory-economy literature also points to a practical skills agenda. If AI-specific oversight roles continue to expand, curricula will need to blend AI literacy, domain translation, ethics, risk governance and product-design thinking. These proposals remain policy implications from the conceptual framework rather than direct conclusions from the present regression, because the panel proxy measures general senior- and middle-management share rather than AI-specific supervisory vacancies.

Artificial intelligence, defined as systems performing tasks requiring human-like intelligence ( Russell & Norvig, 2021), now extends beyond mechanical task substitution to encompass complex decision-making domains. Modern AI architectures incorporate machine learning, natural language processing, and, increasingly, ethical governance modules that require human oversight. This evolution transforms job automation from a simple productivity enhancement to a comprehensive workflow restructuring, where human roles shift from task execution to exception management and ethical calibration ( Brynjolfsson & McAfee, 2022). The automation process consequently creates new hybrid workspaces where humans and machines collaborate through recursive learning loops—systems train humans on new capabilities. In contrast, humans refine algorithmic performance through feedback mechanisms ( Daugherty & Wilson, 2018).

Universal Basic Income (UBI) has emerged as a prominent policy response to technological unemployment, conceptualised as unconditional cash transfers to mitigate the impacts of displacement ( Van Parijs & Vanderborght, 2017). However, empirical evidence increasingly questions its efficacy in addressing structural labour market transitions. The Finnish Basic Income Experiment (2017-2018) revealed negligible employment effects despite improved subjective well-being, with only 3-4% of participants transitioning to new occupations ( Kangas et al., 2019). Similarly, Kenya’s GiveDirectly trial demonstrated temporary consumption smoothing but no sustainable employment pathways ( Banerjee et al., 2020). These outcomes highlight the limitations of UBI as a standalone solution, particularly in developing economies where fiscal constraints limit its scalability. By contrast, AI-supervision roles represent a paradigm shift toward human-machine symbiosis, positioning humans as strategic overseers of automated ecosystems. These roles leverage irreducibly human capabilities—contextual judgment, ethical reasoning, and intercultural interpretation—that currently exceed the capabilities of AI ( Wilson & Daugherty, 2018; Moravec, 1988). The economic value of such symbiosis is evidenced by German manufacturing studies, which show a 22% productivity premium in human-supervised automated systems compared to full automation ( Hirsch-Kreinsen, 2023).

To proxy the potential impact of a Universal Basic Income in contexts where such schemes remain largely experimental, we include “Subsidies and Other Transfers (% of total government expense)” as a continuous measure of fiscal redistributive effort. This indicator captures the share of public expenditure devoted to direct household transfers, approximating the degree to which governments already provide unconditional support akin to a basic income ( Van Parijs & Vanderborght, 2017). Empirical studies of UBI pilots in Finland and Kenya emphasise that cash transfers can improve wellbeing but have had limited effects on employment without complementary labour-market policies ( Banerjee et al., 2020). By controlling for this variable in our model, we account for cross-country and temporal variation in the generosity of social safety nets, ensuring that our estimates of AI exposure and supervisory capacity on unemployment are not conflated with differences in baseline welfare provision. While this measure does not replicate the institutional design of a universal basic income, it captures cross-country and temporal variation in unconditional fiscal support, which is the relevant margin for the present macro-level comparison.

Theoretical frameworks explaining technological displacement require substantial augmentation to account for emerging supervisory economies. While Autor et al.’s (2003) polarisation theory effectively describes the U-shaped employment distribution, it inadequately addresses the generative AI-driven emergence of a “supervisory middle” stratum. Recent Bureau of Labour Statistics data reveals 15% annual growth in AI oversight roles—including algorithm auditors and machine learning validators—with compensation premiums averaging 40% above national medians ( U.S. Bureau of Labor Statistics, 2024). This suggests polarisation models must evolve toward tri-modal frameworks incorporating supervisory layers. Human capital theory ( Becker, 1964) also requires updating to address the velocity of skill obsolescence. Contemporary technological change cycles, which last 3-5 years, outpace traditional education systems, necessitating just-in-time microcredentialing models that the European Union’s AI reskilling initiatives have pioneered ( European Commission, 2023). Economic dependency theory ( Prebisch, 1950) further illuminates the vulnerabilities of the Global South, where limited fiscal space constrains institutional responses to technological disruptions. Middle-income countries like Mexico exhibit automation vulnerability 35% higher than OECD averages, yet invest <0.5% of GDP in AI adaptation infrastructure ( ILO, 2023).

Empirical evidence increasingly validates sectoral divergence in supervisory transitions. Autor and Salomons’ (2021) analysis of Eurostat data demonstrates that financial services automation generates supervisory roles at a rate three times that of manufacturing, primarily in compliance and algorithmic accountability functions. This sectoral granularity proves critical when examining Australian labour transitions through the Internet Vacancy Index (IVI). Post-2020 data reveal an 18% compound annual growth in AI governance positions—particularly “prompt engineers” and “ethical compliance officers”—directly correlating with the adoption of generative AI ( Jobs and Skills Australia, 2025). These roles exhibit distinctive skill hybrids combining technical AI literacy with humanities-informed critical thinking, challenging traditional STEM-focused retraining paradigms ( Davenport & Kirby, 2016).

Cross-national comparisons reveal how institutional architectures shape supervisory transitions. Germany’s “Industrie 4.0” strategy exemplifies coordinated adaptation through dual education systems that integrate technical colleges with industry certification pathways. This approach achieves a 74% success rate in mid-career transitions into automation oversight roles within 18 months ( Hirsch-Kreinsen, 2023). South Korea’s institutional framework similarly demonstrates effectiveness through the mandatory incorporation of AI ethics training into national vocational qualifications since 2022 ( Lee & Choi, 2023). Conversely, liberal market economies exhibit fragmented transitions; U.S. automation hotspots show a 25% wage penalty for displaced workers entering supervisory roles without formal credentials ( U.S. Bureau of Labor Statistics, 2024). Developing economies face compounded challenges, with Brazil’s manufacturing automation yielding only 0.2 supervisory positions per displaced worker due to institutional gaps ( World Bank, 2023).

Behavioural economics provides indispensable insights into human adaptation to supervisory economies. Akerlof and Kranton’s (2000) identity economics framework explains resistance to role transitions in terms of professional identity disruption. Trust dynamics further mediate transitions; Dietvorst et al. (2015) demonstrate that algorithm aversion reduces supervisory effectiveness by 29% when workers perceive AI systems as opaque. This highlights the necessity of human-centred AI design that incorporates explainability features and worker participation in system development ( European Commission, 2021).

From an inclusion standpoint, tracking the proportion of women in middle- and senior-management roles offers more than an equity metric: it serves as a predictor of how effectively an organisation will innovate and adapt to rapid AI uptake. Mixed-gender supervisory teams have been shown to generate more original solutions and temper bias in algorithmic decision-making pipelines ( Østergaard, Timmermans, & Kristinsson, 2011; Dezsö & Ross, 2012). Comparative studies further reveal that, for a given level of automation, business units with higher female representation in supervisory posts experience smoother workforce reallocation and markedly lower "algorithm-aversion," enabling richer human-AI validation loops ( Hoogendoorn, Oosterbeek & Van Praag, 2013; Dietvorst, Simmons & Massey, 2015). Consistent with identity economics theory, inclusive supervisory environments also bolster employee trust and reduce resistance to technological change, thereby mitigating the employment shock of AI diffusion by safeguarding workers’ sense of dignity and agency ( Akerlof & Kranton, 2000).

Institutional frameworks emerge as decisive factors in successful transitions. Effective systems feature regulatory “sandboxes” that allow supervised experimentation, such as Singapore’s AI Verify framework, which reduced implementation risks by 65% ( MAS, 2023). Denmark’s tripartite model shows how collective bargaining agreements can codify supervisory responsibilities, preventing role ambiguity in human-AI collaboration ( Andersen & Ibsen, 2024). Conversely, jurisdictions lacking coherent governance exhibit regulatory arbitrage; nearly 30% of U.S. gig platforms have reclassified supervisory tasks as non-compensable “monitoring activities” ( U.S. Bureau of Labor Statistics, 2024). Developing economies face additional institutional capacity challenges, though Rwanda’s AI governance incubator demonstrates how focused capacity-building can overcome resource constraints ( UNDP, 2023).

This literature reveals three research frontiers that the present panel cannot settle. First, the distributional economics of AI-specific supervisory roles require occupation-level evidence. Second, the psychological toll of continuous system monitoring warrants further study. Third, developing-economy transitions require context-sensitive models that account for informal institutions and fiscal constraints. The supervisory-economy paradigm is therefore best treated as a promising research programme, not as an empirical result already demonstrated by broad management-share data.

A rich interdisciplinary literature converges on the idea that AI does not spell the end of human work, but rather reallocates value to tasks where judgment, contextual insight, and moral reasoning remain indispensable. Human-in-the-loop studies demonstrate that supervised models outperform fully autonomous pipelines on both safety and learning speed, as humans can identify “unknown unknowns” and provide counterfactual feedback that the algorithm cannot infer from data alone ( Dietvorst et al., 2015; European Commission, 2021). Wilson and Daugherty’s collaborative-intelligence taxonomy formalises this complementarity: machines scale, humans steer. In practical terms, supervisor roles range from prompt engineers refining system queries through rapid iteration to algorithmic auditors who stress-test models for fairness and drift, and ethics and compliance officers empowered to veto deployments that breach social thresholds. Such authority is crucial; identity economics experiments demonstrate that workers accept automation only when the residual human task set preserves agency, mastery, and status ( Akerlof & Kranton, 2000).

A dignity-based economic framework remains useful for interpreting the policy stakes of AI adjustment. Sperling (2020) and Sen (1999) both imply that labour-market success should be evaluated not only through aggregate employment, but also through agency, contribution and freedom from humiliation. This normative lens is revisited in the discussion, but it is not part of the econometric identification strategy.

Operationalising this lens requires more granular evidence than is available in the present panel. Future Australian work could use vacancy microdata, credential data and firm-level AI adoption measures to test whether AI-governance curricula, risk-audit roles and domain-translation roles generate measurable employment, trust and well-being gains.

## Methodology

To explore preliminary patterns and potential interactions between key variables, we plotted a series of scatter plots using the panel data. These included the relationship between the AI Exposure Index and the share of senior and middle management (SMMS), as well as other control variables such as R&D expenditure, public transfers, and GDP per capita. The scatter plot between SMMS and AI Exposure revealed a moderately dispersed distribution without strong linearity, suggesting that their interaction in the regression specification requires further examination. These visualisations helped motivate the inclusion of interaction terms and non-linear (squared) specifications in the econometric model, particularly to test whether supervisory capacity moderates the impact of AI exposure on unemployment outcomes.

**Figure 1.**
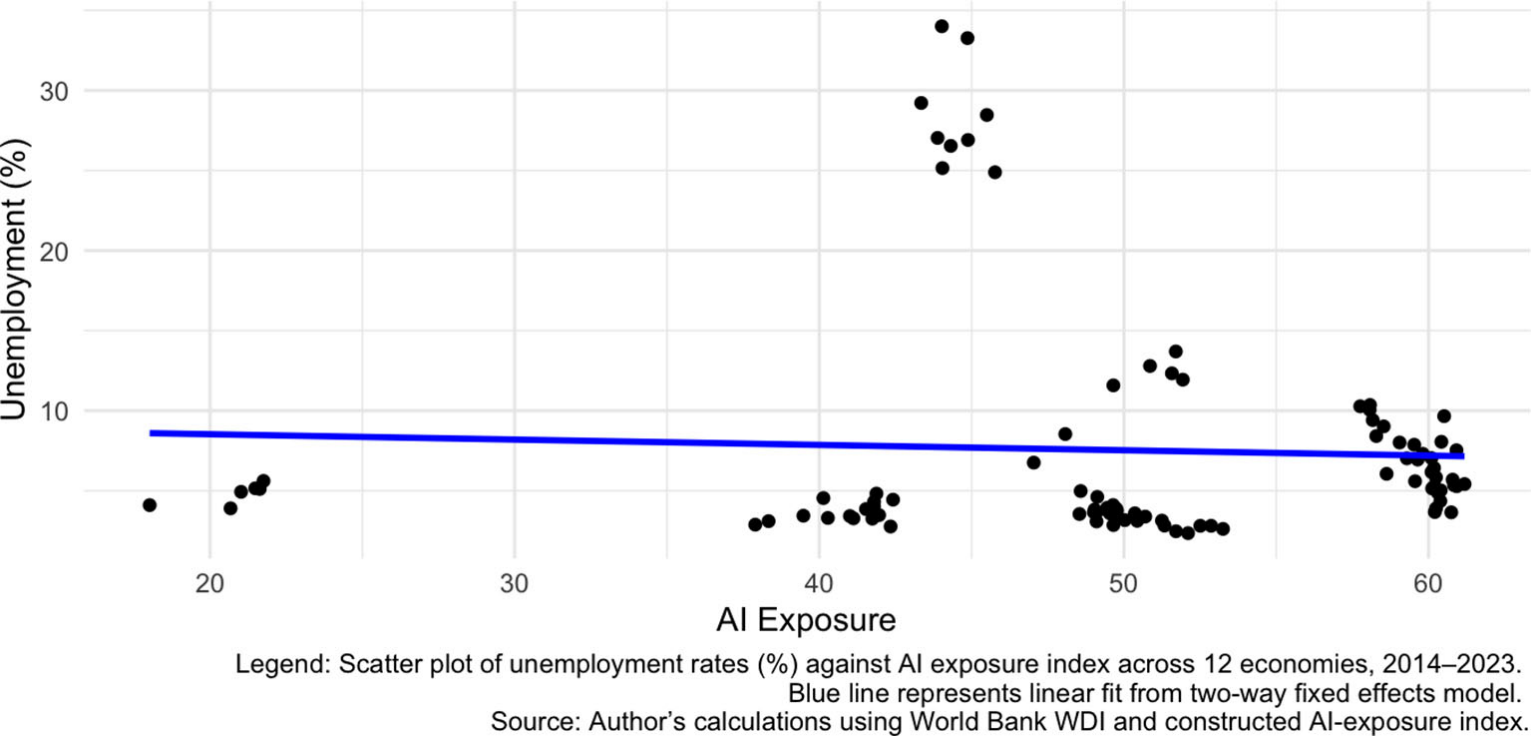
AI Exposure vs. Unemployment Rate across 12 Economies (2014–2023). Source: Author’s calculations based on the AI exposure index and World Bank unemployment data (2014–2023), created in R.

**Figure 2.**
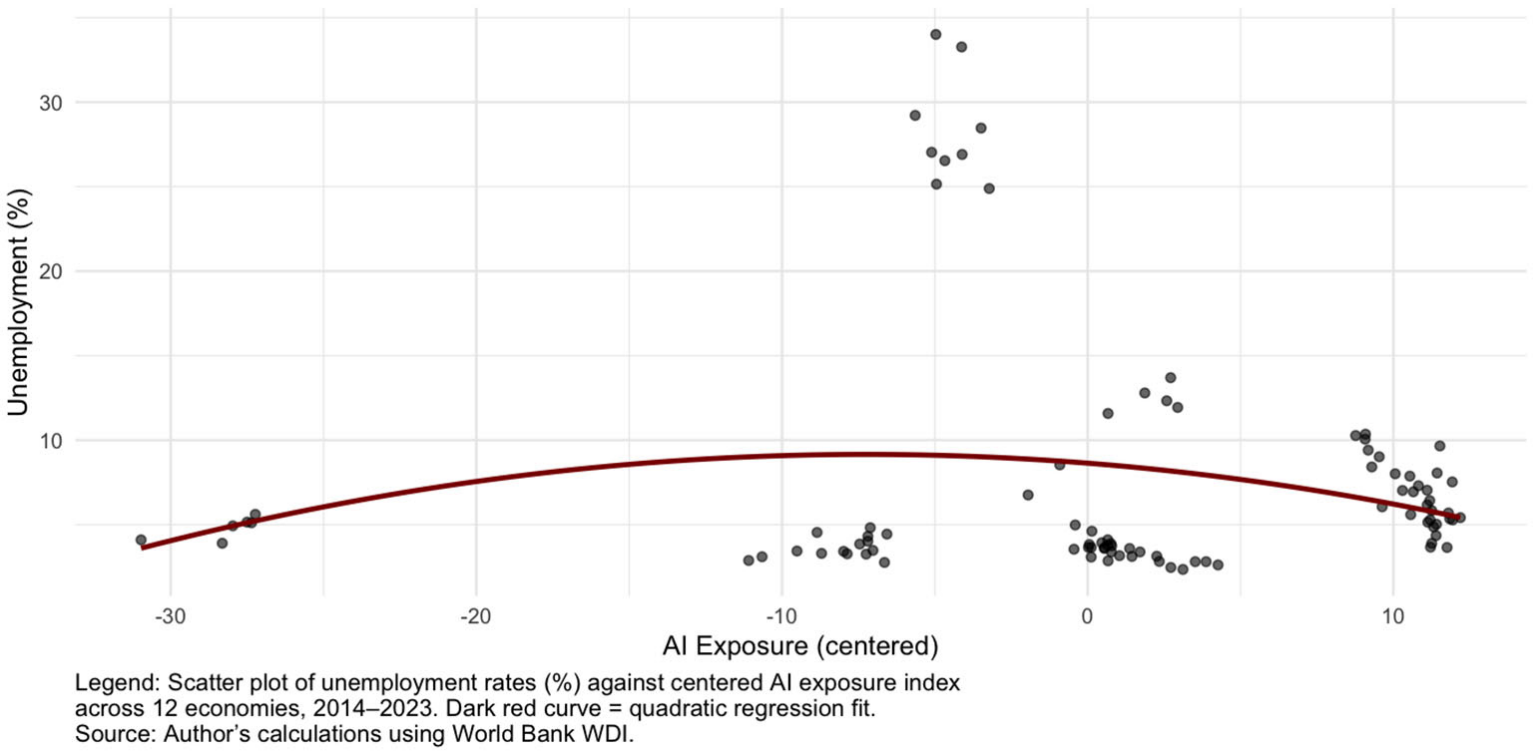
Relative AI Exposure vs. Unemployment Rate across 12 Economies (2014–2023). Source: Author’s calculations based on the AI exposure index and World Bank unemployment data (2014–2023), created in R. Relative AI Exposure” is computed by subtracting the overall sample mean of AI exposure from each observation, so that values above zero indicate economies–years with AI adoption above the 2014–2023 average, and values below zero indicate levels below that mean.

**Figure 3.**
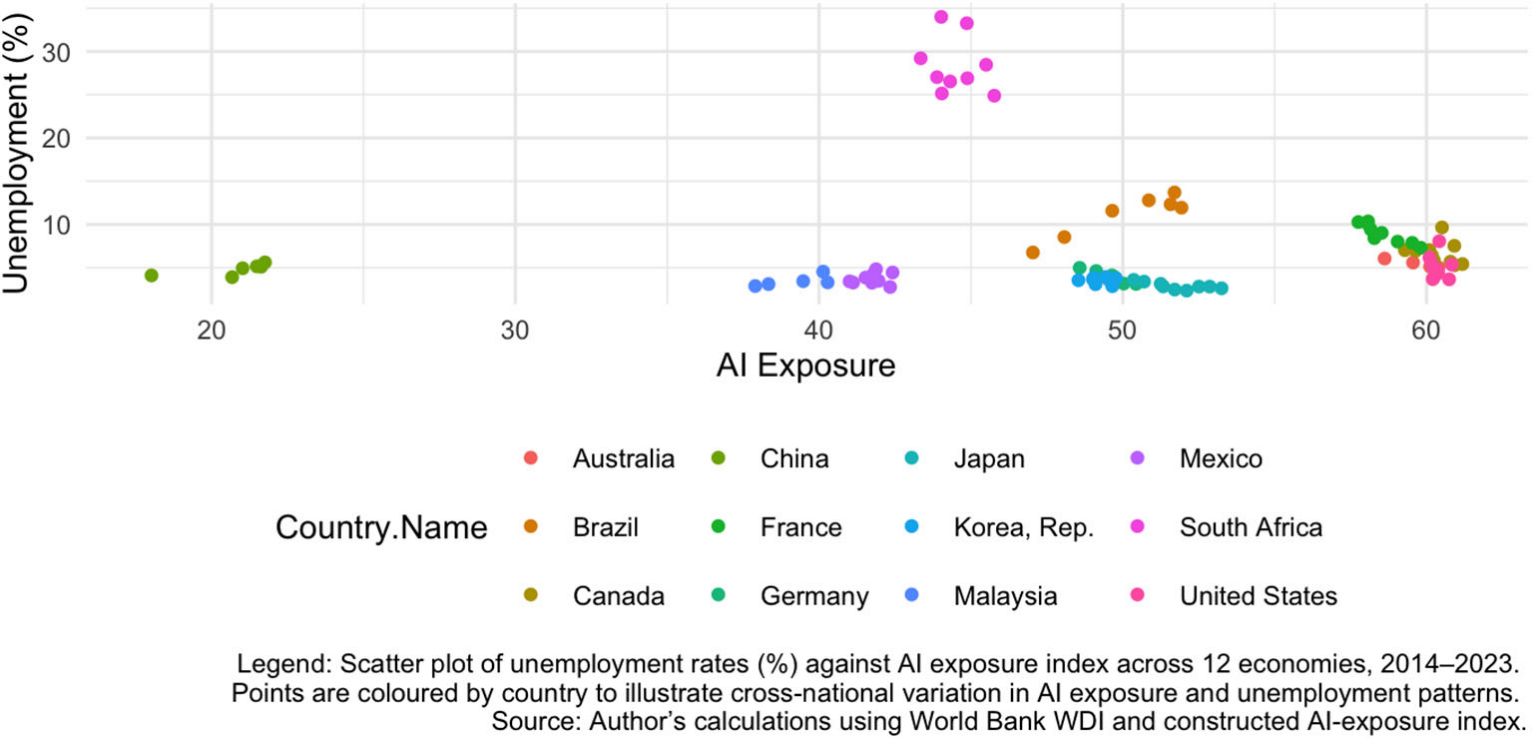
Country-Level Unemployment vs. AI Exposure (2014–2023). Source: Author’s calculations using the sector-weighted AI exposure index and World Bank unemployment data for 12 economies (2014–2023). Each point represents one Country–year observation, with colours distinguishing individual countries. Created in R.

**Figure 4.**
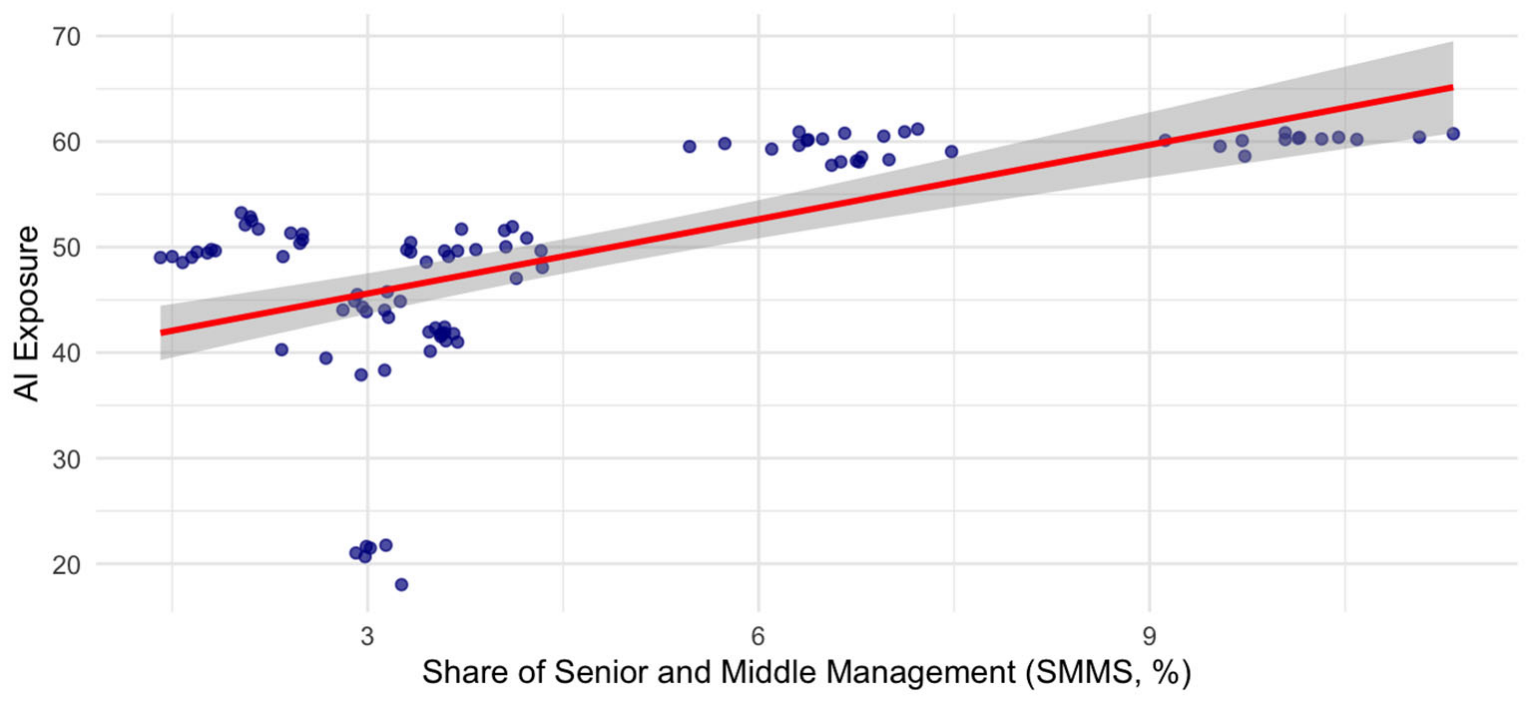
Relationship between Supervisory Share (SMMS% %) and AI Exposure (2014–2023). Source: Author’s calculations based on the sector-weighted AI exposure index and the share of senior- and middle-management roles (SMMS% %) drawn from national labour-force statistics (2014–2023) and created in R.

**Figure 5.**
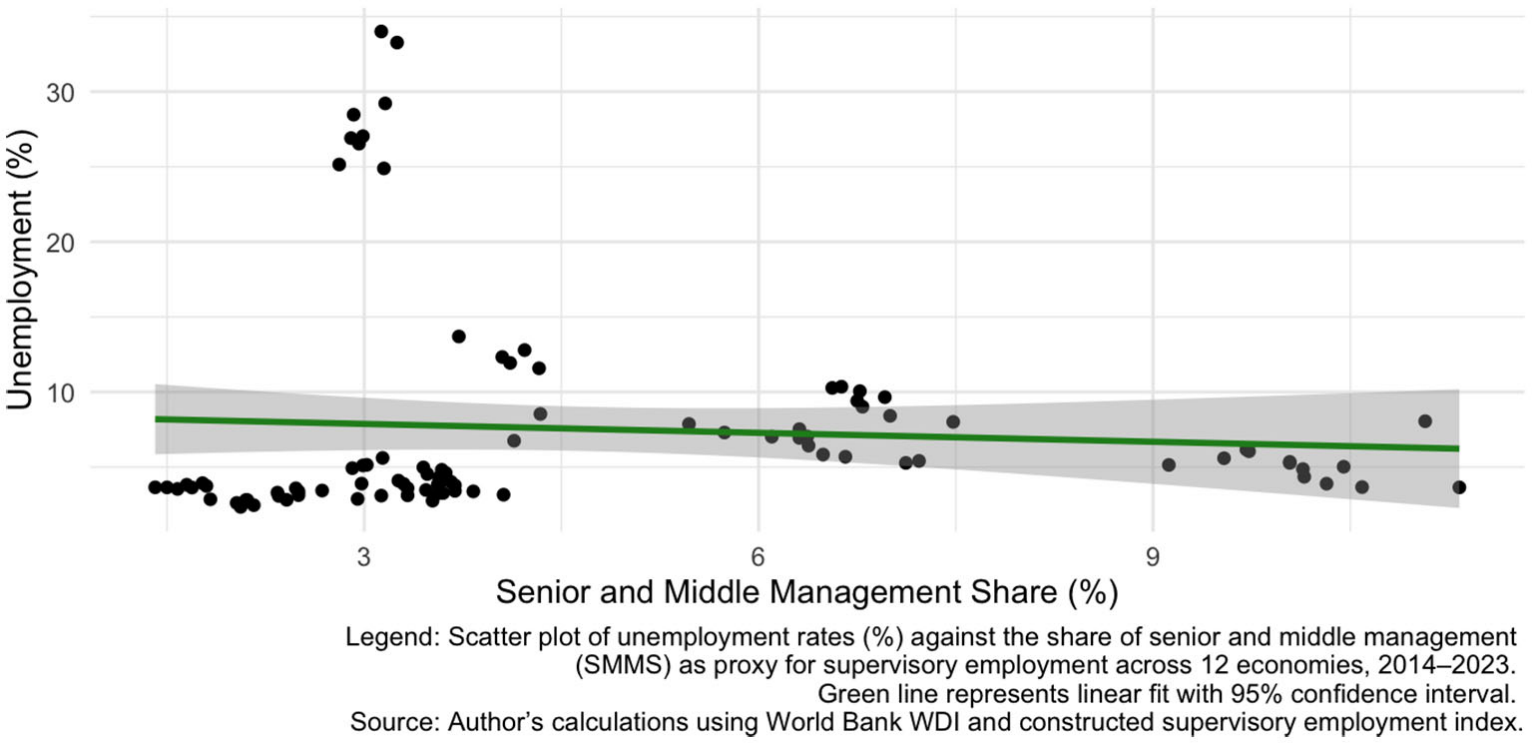
Senior and Middle Management Share vs. Unemployment Rate (2014–2023). Source: Author’s calculations based on national labour-force statistics for the share of senior- and middle-management roles (SMMS% %) and World Bank unemployment data across 12 economies (2014–2023) and created in R.

Figures 1; 2; 3; 4 and 5 summarise the key empirical relationships between AI exposure, supervisory employment and unemployment across the 12-country panel.

Figure 1 depicts a simple bivariate scatter plot of aggregate AI exposure against unemployment, revealing only a shallow, statistically insignificant linear slope and suggesting that a naïve linear specification may mask important non-linearity. Figure 2 replots the same relationship after mean-centring the exposure variable and fitting a quadratic trend; an inverted-U emerges, with unemployment declining once relative exposure reaches the upper end of the observed distribution. Figure 3 disaggregates the raw data by country, showing that the aggregate curve conceals distinct clusters: low-exposure/low-unemployment economies, mid-exposure/high-unemployment cases and high-exposure/moderate-unemployment OECD members. Figures 4 and 5 describe the managerial-share proxy. They should be read cautiously: SMMS captures broad senior- and middle-management employment, not AI-specific oversight vacancies. Together, the visuals motivate the preferred quadratic specification while showing that the supervisory-economy mechanism remains only indirectly proxied in the present data.

The empirical strategy rests on a balanced panel of twelve countries—six developed and six developing—whose contrasting labour-market structures are summarised in Table 1. The methodology proceeds in three steps: (i) construction of an aggregate AI-exposure index that weights occupation-level AIOE scores by each country’s sectoral employment shares; (ii) use of senior- and middle-management share as a broad proxy for organisational oversight capacity, with explicit recognition that it does not measure AI-specific governance labour; and (iii) estimation of two-way fixed-effects models that test the linear and quadratic association between AI exposure and unemployment after controlling for income, public transfers and R&D effort. This framework is appropriate for testing a macro-level association and an exploratory moderation effect, but not for making strong causal claims about the emergence of AI-governance occupations.

**Table 1.** Country classification by development level and stylised automation exposure).

Country	Development group	Stylised automation exposure ^1^
United States	Developed	**High** – very large services sector, pervasive AI adoption in finance, retail, logistics
Germany	Developed	**High**–world–class manufacturing with advanced robotics plus data-intensive services
Japan	Developed	A high–aging workforce drives intensive deployment of industrial and service-sector automation.
Australia	Developed	**Medium** – commodity extraction lowers aggregate exposure, but services are heavily digitalised.
United Kingdom	Developed	**High**–finance, media and professional services dominate the employment mix
Canada	Developed	**Medium** – diversified economy; strong services offset by sizeable resource and primary sectors
China	Developing	**Medium** – rapid robot diffusion in export manufacturing, but still, large, low-tech services share
India	Developing	**Low**–employment concentrated in agriculture and informal services; limited formal automation
Brazil	Developing	**Low**–high share of agriculture and basic services; uneven industrial automation
South Africa	Developing	**Low** services dominate, but mostly low-tech, with modest AI penetration
Indonesia	Developing	**Low**–agriculture and low-skill manufacturing keep aggregate exposure subdued.
Mexico	Developing	**Medium** – export-oriented manufacturing is increasingly automated, yet agriculture and informal services remain sizeable.

By the author.

Source: International Federation of Robotics (2024, 2022); Jobs and Skills Australia (2025); World Economic Forum (2023, 2025); McKinsey & Company (2017); World Bank – WDI (2023).

^1^ Classification is based on the sector-weighted AI-exposure index constructed in this paper: “High” = top third of baseline index, “Medium” = middle third, “Low” = bottom third.

Table 1 below presents the 12 selected countries, grouped into developed economies and developing/emerging economies, along with an approximate qualitative assessment of their automation exposure, categorised as high, medium, or low. This categorisation considers factors such as industrial structure, technology adoption rates, investment in artificial intelligence, and workforce composition, providing a practical foundation for analysing AI-driven labour market transformations.

The balanced panel now comprises twelve economies—six developed and six developing—observed annually from 2014 to 2023. Developed members (the United States, Germany, Japan, Australia, the United Kingdom, and Canada) represent high-income OECD countries with sophisticated labour markets. Developing members (China, India, Brazil, South Africa, Indonesia, Mexico) provide contrast in occupational structure and technological diffusion. All macro variables are drawn from the World Development Indicators.

For each country-year, we build a sector-weighted AI-exposure index, AIExpc,t, using constant occupational AIOE benchmarks obtained from the SOCAIOE database and sectoral employment shares from the World Development Indicators. The country-year index is computed as the employment-share-weighted average of sectoral exposure scores, then z-standardised across the estimation panel so that one unit represents one sample standard deviation of exposure. This construction means that estimated coefficients should be interpreted within the observed country-year support rather than as universal technology elasticities.

We construct the Share of Senior-and-Middle-Management (SMMS) as the percentage of workers classified in senior and middle management over total employment in each country-year, drawing on harmonised ILOSTAT and World Bank labour-force series where available. This variable is retained because it provides the most comparable cross-country indicator of broad organisational hierarchy. Its limitation is central to the revised interpretation: most senior and middle managers are not AI-governance specialists, so SMMS is a noisy stock measure of general managerial intensity rather than a flow measure of new AI-specific oversight jobs. Missing occupational observations are handled transparently through documented interpolation or nearest-year rules in the replication file, and results should be read as exploratory where SMMS availability is incomplete.

Supervisory capacity is therefore proxied by “Employment in senior and middle management (% of total employment)” (SupEmpc,t/SMMS). This proxy should not be interpreted as a direct measure of AI oversight labour. To capture broad income-support effort, we include Subsidies and Other Transfers (% of Total Government Expense), a World Bank indicator measuring the share of public expenditure devoted to subsidies and social transfers. Denoted Transfc,t, this variable controls for cross-country and temporal variation in fiscal redistribution. The core two-way fixed-effects specification is

Unempc,t=α+βAIExpc,t+δSupEmpc,t+ϕFemaleMgrc,t+θ(AIExpc,t×SupEmpc,t)+ψTransfc,t+γ1lnGDPpcc,t+γ2RDc,t+μc+λt+εc,t

### Variables

Unempc,t= Unemployment rate – the percentage of the total labour force that is unemployed in country ccc and year *t*.

βAIExpc,t= Aggregate exposure to artificial intelligence – a country-level index constructed by weighting occupational AI exposure (AIOE) by the employment shares across sectors, for country ccc in year *t*.

ϕFemaleMgrc,t= Percentage of managerial positions (senior and middle management) held by women in country c and year *t*. Serves as a control for gender diversity in supervisory roles, which may affect how effectively a workforce adapts to AI-driven changes.

δSupEmpc,t= General managerial share – the percentage of total employment in senior and middle management in country *c* and year *t*; this is a coarse proxy for organisational oversight capacity, not a direct measure of AI-specific supervisory labour.

θ(AIExpc,t×SupEmpc,t)= Interaction term between AI exposure and general managerial share – tests whether broad managerial intensity moderates the AI-unemployment association; it does not adjudicate the existence of AI-specific oversight vacancies.

ψTransfc,t= Subsidies and other transfers (% of total government expense), a proxy for the generosity of the fiscal safety net in country *c* and year *t*.

γ1lnGDPpcc,t= Log of GDP per capita (in constant 2021 international dollars, PPP-adjusted), a control variable to account for income level.

γ2RDc,t= Research and development expenditure – measured as a percentage of GDP, representing national investment in innovation and technological capacity.

μc= Country fixed effects – controls for unobservable country-specific factors that do not vary over time.

λt = Time fixed effects – controls for global or time-specific shocks that affect all countries similarly each year.

εc,t= Error term – the idiosyncratic error for country *ccc* in year *t*.

We first ran a two-way fixed-effects regression on the balanced 2014–2023 panel. In the linear model, higher AI exposure is associated with lower unemployment (−1.47 percentage points per standardised AI unit, p ≈ 0.04), but this estimate should be treated as a diagnostic baseline because the later quadratic model shows that the association is not constant across the exposure distribution. The broad managerial-share proxy is only marginally associated with lower unemployment (−2.12 percentage points, p ≈ 0.07), and the AI × management interaction is not statistically significant. These results do not support a strong claim that oversight roles protect employment; rather, they motivate the non-linear specification and show that general managerial intensity is, at best, an imprecise moderator. The positive R&D coefficient is also interpreted cautiously, since it may reflect transitional displacement, reverse causality or omitted innovation-cycle factors.

**Table 2.** Two-way Fixed-Effects Linear Model: Unemployment, AI Exposure, Supervisory Employment and Controls (2014–2023).

Regressor	Coefficient	Std. error	p-value	Interpretation
**AI Exposure (AIExp)**	−1.47 *	0.69	0.043	Linear baseline only; effect is non-linear in the preferred model
**Supervisory employment (SupEmp)**	−2.12 ^†^	1.14	0.071	Broad managerial share is only marginally associated with unemployment
**AIExp × SupEmp**	+0.02	0.02	0.170	Interaction is not significant; no identified protective mechanism
**Subsidies & transfers (% expense)**	+0.14	0.09	0.154	Fiscal transfers have no apparent effect
**ln GDP per capita (PPP)**	−68.45 ***	13.88	0.000	Richer economies exhibit markedly lower unemployment
**R&D expenditure (% GDP)**	+5.42 *	2.39	0.036	Positive association; interpret cautiously because timing and omitted factors may matter

Fixed effects: country & year  Clusters: Country.

Observations: 66  R ^2^ (within): 0.988.

Source: Author’s estimates from a two-way fixed-effects panel regression covering 12 economies for the years 2014–2023.

*** p < 0.001;

** p < 0.01;

* p < 0.05;

^†^ Indicates marginal significance (p < 0.10). Two-way fixed effects include country and year; standard errors are clustered at the country level.

We first ran a two-way fixed-effects regression on the balanced 2014–2023 panel. In the linear model, higher AI exposure is associated with lower unemployment (−1.47 percentage points per standardised AI unit, p ≈ 0.04), but this estimate should be treated as a diagnostic baseline because the later quadratic model shows that the association is not constant across the exposure distribution. The broad managerial-share proxy is only marginally associated with lower unemployment (−2.12 percentage points, p ≈ 0.07), and the AI × management interaction is not statistically significant. These results do not support a strong claim that oversight roles protect employment; rather, they motivate the non-linear specification and show that general managerial intensity is, at best, an imprecise moderator. The positive R&D coefficient is also interpreted cautiously, since it may reflect transitional displacement, reverse causality or omitted innovation-cycle factors.

Because the linear model compresses different exposure regimes into one slope, we re-specified AI exposure with a quadratic term, β2(AIExpc,t)^2 , to capture curvature. This specification is the preferred model because it tests whether the unemployment association changes sign across the observed distribution.

### Modelling non-linear effects of AI exposure: Quadratic specification

We assembled a balanced panel of 12 economies, observed annually from 2014 to 2023 (96 country–year observations after list-wise deletion). For each country-year, we combine sectoral employment shares from the World Development Indicators with occupation-level automation scores to construct an aggregate AI-exposure index, AIExpc,t. To allow for non-linearity, the index is first demeaned,

AIExpc,t∗=AIExpc,t−AIExp,

Moreover, its square, (AIExpc,t∗)2, is included as an additional regressor. Supervisory capacity is proxied by the share of senior- and middle-management employment (SMMS). Further controls comprise the log of GDP per capita, the ratio of public expenditure on subsidies and transfers, and R&D expenditure as a percentage of GDP. Two-way fixed effects absorb time-invariant country heterogeneity (μc) and common year shocks (λt), and standard errors are clustered at the country level to account for arbitrary serial correlation within countries.

The preferred specification is estimated with two-way fixed effects and clustered standard errors:

Unemploymentc,t=β1AIExpc,t∗+β2(AIExpc,t∗)2+β3SMMSc,t+β4(AIExpc,t∗×SMMSc,t)+β5lnGDPpcc,t+β6Transfc,t+β7RDc,t+μc+λt+εc,t.

Estimates reveal a positive but insignificant linear AI term and a negative, statistically significant quadratic term (β2 ≈ –0.052, p ≈ 0.03), consistent with an inverted-U pattern: unemployment rises at low-to-moderate AI exposure and declines only toward the upper end of the observed exposure distribution. The interaction between AI exposure and SMMS is negative but not statistically significant, so the model does not identify a protective supervisory-economy mechanism. Higher GDP per capita is associated with lower unemployment, but the large coefficient should be interpreted within the narrow within-country support of the fixed-effects model and checked in robustness work using standardised effects and marginal-effect plots.

When alternative covariates were tested, none overturned the concave AI finding. Introducing the female share of senior and middle management trimmed the usable sample and produced imprecise estimates; omitting it improved within-fit and left the AI coefficients materially unchanged. Substituting contemporaneous income with a one-year lag yielded similar AI parameters, while lagged income lost statistical significance. Fiscal transfers remained small and insignificant across variants. The positive, marginally significant R&D term turned insignificant when lagged, which is consistent with either short-run displacement or timing and omitted-variable concerns. Collectively, these checks suggest that the inverted-U association is robust, while the mechanisms behind income, R&D and supervisory capacity require further testing.

## Results

The two-way fixed-effects estimations, with heteroskedasticity-robust standard errors clustered at the country level, yield a consistent narrative across functional forms. In the preferred quadratic specification, the centred linear AI-exposure term is economically small and imprecise ( $\hat{\beta }$ = 0.174, SE = 0.436, p = 0.69). In contrast, the squared term is negative and statistically significant ( $\hat{\beta }$ = –0.0516, SE = 0.0228, p = 0.03), implying a concave unemployment-technology locus. The implied turning point occurs at approximately 1.69 standard deviations above the sample mean of exposure; this value should be read as a within-sample threshold, not a universal adoption benchmark. The supervisory-workforce share (SMMS) carries an insignificant standalone coefficient ( $\hat{\beta }$ = 0.503, p = 0.33) and its interaction with exposure is likewise non-significant ( $\hat{\beta }$ = –0.0455, p = 0.34). Thus, broad managerial depth does not materially modulate the concave pattern once country and year fixed effects are partialled out. The GDP-per-capita coefficient is large ( $\hat{\beta }$ = –33.55, p = 0.03), so the substantive interpretation should rely on marginal effects over the observed within-country range and standardised robustness checks rather than on extrapolated doublings of income. Contemporaneous public transfers are not statistically significant (p = 0.12), while R&D outlays are marginally positive (p = 0.08) and should be interpreted cautiously.

**Table 3.** Two-way Fixed-Effects Quadratic Model: Unemployment, AI Exposure, Supervisory Share and Controls (2014–2023).

Term	Coefficient (cluster s.e.)	p-value	What it tells us
**AIExp_c**	0.174 (0.436)	0.69	Small, imprecise linear component.
**AIExp** ^**2**^	**–0.0516 (0.0228)**	**0.03**	Significant concave curvature (inverted-U).
**SMMS**	0.503 (0.514)	0.33	A standalone supervisory share is not decisive.
**AIExp × SMMS**	–0.0455 (0.0470)	0.34	Not significant; broad management share does not identify AI-specific oversight labour.
**ln GDPpc**	**–33.55 (14.99)**	**0.03**	Large negative fixed-effects coefficient; interpret within observed support.
**Transfers**	0.092 (0.059)	0.12	Transfers do not offset job loss contemporaneously.
**R & D**	1.69 (0.92)	0.08	Marginal positive association; mechanism remains uncertain.

Source: Author’s estimates in R from a two-way fixed-effects panel regression using annual data for 12 economies (2014-2023). Standard errors are clustered at the country level. Variables: unemployment rate (dependent); centred AI exposure (AIExp_c) and its square; share of senior- and middle-management positions (SMMS); their interaction; log GDP per capita; public transfer spending (% GDP); and R&D expenditure (% GDP).

Bold coefficients indicate statistical significance at the 5% level (p < 0.05). Two-way fixed effects include country and year; standard errors are clustered at the country level.

The linear specification acts as a robustness probe: omitting the quadratic term renders the AI-exposure coefficient significantly negative ( $\hat{\beta }$ = –1.47, p = 0.043), but this result is sensitive to high-exposure, high-income economies and weakens once curvature is admitted. Supervisory intensity is weakly beneficial in the linear model ( $\hat{\beta }$ = –2.12, p = 0.071), yet the interaction remains non-significant (p = 0.17). Overall, the econometric evidence supports a concave association between AI exposure and unemployment, tempered by national income, with broad managerial layering exhibiting, at most, second-order and statistically uncertain effects.

## Conclusions

The evidence reveals an inverted-U association between national AI exposure and unemployment: modest automation pressures coincide with higher joblessness, but toward the upper end of the observed exposure distribution the marginal association turns negative. This pattern is consistent with models in which automation first displaces routine labour and later complements new tasks, products and organisational forms. The paper’s strongest empirical contribution is therefore the concave AI-unemployment result. By contrast, the broad managerial-share proxy neither reduces unemployment on its own nor significantly moderates the AI effect in the preferred model. This means the present data do not support a causal claim that AI-specific oversight roles protect employment. They show only that general managerial intensity, as measured here, is not a statistically reliable buffer.

Taken together, the findings support a balanced narrative from the wider literature: AI adoption can initially widen labour-market fractures but may re-equilibrate when complementary tasks and productivity gains scale. However, the non-significant interaction between AI exposure and SMMS requires restraint. Policies such as AI oversight corps, algorithmic-risk fellowships or prompt-engineering programs remain conceptually plausible, but they are not direct implications of the moderation estimate. They should be framed as policy experiments justified by the literature and by emerging vacancy evidence, not as outcomes proven by this panel.

The study has three important limitations. First, the panel ends in 2023, so it captures the pre-generative-AI era and only the earliest phase of foundation-model diffusion. Second, SMMS is an economy-wide management-stock measure and is too coarse to test the growth of AI-governance, algorithmic-risk, model-monitoring or prompt-engineering jobs. Third, coefficient magnitudes for controls such as log GDP per capita should be interpreted within observed support and stress-tested through standardised effects, marginal-effect plots and alternative functional forms. A more conclusive assessment will require higher-frequency data, a country-year availability table, a deposited constructed panel and vacancy-level measures of AI-specific oversight labour.

### Policy implications

The policy implications drawn from this analysis should be interpreted as conditional and forward-looking. The regression evidence supports caution about early displacement and highlights the importance of income capacity, but it does not show that simply increasing the number of managers will reduce unemployment. Governments should therefore treat AI Oversight Corps, AI Safety Engineer fellowships and prompt-engineering or data-translation programs as targeted pilots to be evaluated with vacancy-level and worker-level evidence. The aim is not to expand management layers generically, but to build measurable AI-governance capability where high-risk systems are deployed and where displaced workers can realistically move into complementary tasks.

AI may generate occupations that are difficult to imagine ex ante, but the transition’s human costs and distributional outcomes remain contingent on political choices rather than technological determinism. Without deliberate institutional steering toward human-AI collaboration, robust skills ecosystems and inclusive governance, the productivity gains from AI may arrive with unnecessary displacement and inequality. The employment future remains unwritten; this paper shows one macro pattern to investigate further, not a settled verdict.

The next step is to develop a real-time AI Jobs Barometer. Such a tool should track vacancy titles, task descriptions, salary trends, credential requirements and sectoral diffusion for AI-governance, algorithmic-risk, model-monitoring, prompt-engineering and data-translation roles. This would directly address the proxy limitation in the present paper and allow future work to test whether AI-specific oversight labour grows quickly enough, and inclusively enough, to offset displacement.

## Data Availability

All raw inputs used in this study are derived from publicly available secondary sources. The AI exposure index was constructed using occupational AI exposure scores from the SOCAIOE database and sectoral employment shares from the World Development Indicators ( https://data.worldbank.org/indicator). Supervisory employment data, representing senior and middle management as a share of total employment, were retrieved from ILOSTAT and World Bank labour-force series ( https://www.ilo.org/ilostat). Other macroeconomic controls, including GDP per capita (PPP, constant 2021 international dollars), R&D expenditure (% GDP), transfers (% government expense) and unemployment, were sourced from the World Bank, ILO and OECD databases. For full reproducibility, the resubmission should include the constructed country-year panel, the AI-index construction script, missingness rules, and a country-year availability table as source data or extended data. No primary data collection was conducted, and there are no restrictions or embargoes on the raw data. Because the empirical contribution depends on the constructed AI-exposure panel, reproducibility is strongest when the exact cleaned dataset and code are deposited alongside the article rather than requiring readers to reconstruct the pipeline from raw sources alone. All materials for this study are derived entirely from publicly available secondary sources, and therefore, no primary data instruments (e.g., questionnaires, consent forms, interview guides) were created.
