# Supplementary material for: The Concave Relationship Between AI Exposure and Unemployment: Reframing the Supervisory Economy as an Exploratory Moderation Test
Source: F1000Res. 2026 May 14;14:1211. Originally published 2025 Nov 5. [Version 3] doi: 10.12688/f1000research.168512.3 (PMC13264688; doi:10.12688/f1000research.168512.3)
Supplement: Peer review history

# Reviewer report: Reviewer response for version 3

**DOI:** 10.5256/f1000research.200892.r485016

**Published:** 2026-06-17

**Authors:** Fiyinfoluwa Giwa

**Affiliations:** 1 University of South Africa, Pretoria, South Africa

The manuscript examines an important and contemporary research question concerning the labour-market implications of artificial intelligence and seeks to contribute to the emerging literature by investigating whether the relationship between AI exposure and unemployment is non-linear. The authors have invested considerable effort in developing the conceptual framework, assembling a cross-country panel dataset, and responding to earlier reviewer feedback. The revised manuscript is more measured in its interpretation of the supervisory economy concept and appropriately recognizes several of its own empirical limitations. The exploration of a concave AI-unemployment relationship represents a potentially valuable contribution to the literature. However, while the study has merit and addresses a topic of significant scholarly and policy interest, there remain several important concerns relating to the conceptual framing, variable construction, methodological transparency, and interpretation of the findings that should be addressed before the manuscript can be considered sufficiently robust.

**Title and abstract:**

 The title is now more accurate because the paper mainly tests a concave AI exposure and unemployment relationship, not the full “supervisory economy” mechanism.

**Introduction:**

 The introduction is timely and relevant, but it still promises more than the empirical design can deliver. The paper discusses AI-supervisory jobs, UBI, and labour-market transitions, yet the actual model only uses broad senior and middle management share as a proxy. This creates a gap between the conceptual ambition and the empirical test. The authors should narrow the framing and avoid language suggesting that displaced workers can realistically be absorbed into AI-supervisory roles unless they provide direct evidence. Another option could be to justify the use of broad senior and middle management share as a proxy.

**Literature review:**

 This section too long and sometimes reads like a general essay on AI, automation, UBI, gender, ethics, and human dignity. Many parts are relevant, but not all are directly linked to the regression model. The review should be reorganized around three clear strands: AI exposure and unemployment, non-linear labour-market effects, and supervisory/managerial capacity. The UBI and dignity discussions should be shortened or moved to policy discussion.

**Theory and hypotheses:**

 The theories are relevant, especially employment polarization and human capital theory. However, economic dependency theory is not strongly integrated into the empirical model. The hypotheses are now more modest, which is good, but H2 remains weak because SMMS does not measure AI-specific supervision. The paper should state clearly that this is a proxy validity limitation, not just a data limitation.

**Methodology:**

 This is the weakest section. The paper uses a 12-country panel from 2014 to 2023 and estimates two-way fixed effects, but the construction of the AI exposure index is not sufficiently reproducible. The authors should provide the exact formula, data sources, sector weights, interpolation rules, missing data treatment, and country-year availability table (this can be in the appendix). The paper also reports 96 observations after deletion in one place, but Table 2 reports 66 observations, which needs explanation.

**Model specification:**

 The quadratic model is reasonable, but it is not enough. The authors should test robustness using alternative specifications, such as excluding outlier countries, using lagged AI exposure, standardizing all variables, adding labour-market controls, and checking whether the inverted-U is driven by high-income countries. With only 12 countries, clustered standard errors may also be fragile.

**Results:**

 The main result is interesting: the squared AI exposure term is negative and significant, suggesting an inverted-U relationship. However, the linear AI term is insignificant, SMMS is insignificant, and the interaction is insignificant. Therefore, the paper’s real finding is only the concave association, not evidence for a supervisory economy. The authors should avoid implying that supervisory roles explain the decline in unemployment at high AI exposure.

**Discussion and policy implications:**

 The discussion is more cautious than earlier versions, but policy recommendations such as AI oversight corps and prompt-engineering programs still go beyond the evidence. These should be framed as possible policy experiments, not direct implications of the regression. The authors already acknowledge that vacancy-level data would be needed, and this point should be made stronger.

Is the work clearly and accurately presented and does it cite the current literature?

Yes

If applicable, is the statistical analysis and its interpretation appropriate?

Yes

Are all the source data underlying the results available to ensure full reproducibility?

Partly

Is the study design appropriate and is the work technically sound?

Partly

Are the conclusions drawn adequately supported by the results?

Partly

Are sufficient details of methods and analysis provided to allow replication by others?

Partly

Reviewer Expertise:

Technological Economics, Labour Market,  Energy

I confirm that I have read this submission and believe that I have an appropriate level of expertise to confirm that it is of an acceptable scientific standard, however I have significant reservations, as outlined above.

# Reviewer report: Reviewer response for version 3

**DOI:** 10.5256/f1000research.200892.r487422

**Published:** 2026-06-13

**Authors:** Rossi Passarella

**Affiliations:** 1 Computer Science, University of Sriwijaya, Inderalaya, Sumatera-selatan, Indonesia

This paper examines the relationship between aggregate exposure to Artificial Intelligence (AI) and unemployment rates using balanced panel data from 12 countries (6 developed and 6 developing) over the period 2014–2023.

 The main empirical contribution of this study is the discovery of a reverse-U-shaped (concave) non-linear relationship between AI exposure and unemployment. At low to moderate levels of AI exposure, unemployment tends to rise (indicating an initial substitution effect/labor displacement). However, after passing a certain inflection point (identified at 1.69 standard deviations above the sample mean), the relationship turns negative—meaning unemployment begins to decline in countries with very high AI exposure. The authors attribute this re-equilibrium to a theory in which AI ultimately creates new tasks, boosts productivity, and sparks new forms of organization.

 Additionally, this article tests a policy concept called the “supervisory economy”, an idea where workers displaced by AI can be redirected to become supervisors or auditors of those AI-powered systems. To test this mechanism at the macro level, the authors use the Share of Senior and Middle Management (SMMS) as a moderating variable. However, statistical tests show that neither the SMMS variable nor its interaction term with AI exposure is statistically significant, thus failing to empirically prove that general managerial capacity acts as a shield for the labor market against AI disruption.

 1. Is the work clearly and accurately presented and does it cite the current literature?

 My choice: **Partly**

**Justification:**

*From my perspective after reading it, I believe this manuscript has successfully presented a very rich and up-to-date body of literature on labor polarization and the impact of artificial intelligence. However, I observe a significant construct mismatch in the presentation of its main argument.*

*This mismatch lies in the theoretical narrative built upon specific ideas regarding “AI-supervisory roles” (such as prompt engineers and algorithm auditors), whereas the empirical analysis employs a very crude macro-level proxy: the overall share of senior and middle management (Share of Senior and Middle Management / SMMS).*

*This mismatch risks confusing readers/the audience regarding what is actually being statistically tested.*

 2. Is the study design appropriate and is the work technically sound?

 My choice: **Partly**

**Justification:**

*The author employs a Two-Way Fixed Effects (TWFE) regression with a quadratic model, which I believe is technically sound; nonetheless, I believe the study design is slightly inadequate in terms of verifying the main mechanism (supervisory economy).*

*This is because the SMMS variable only reflects the overall stock of managers across multiple businesses (including retail or traditional manufacturing managers), not the flow of new job openings related to AI supervision.*

*As a result, I believe the interaction tests in this design do not have sufficient construct validity to address the authors' main premise.*

**3. Are sufficient details of methods and analysis provided to allow replication by others?**

 My choice : **Partly**

**Justification:**

*The authors have not provided:*

1. *Detailed steps for calculating transformations and weighting in the construction of the AI exposure index.*
2. *A country-year availability table, given the interpolation of missing management data.*
3. *R- code is not given*

**4. If applicable, is the statistical analysis and its interpretation appropriate?**

 My choice: **Partly**

**Justification:**

*The results of the quadratic model estimation indicate a statistically significant concave relationship (in Table 3: AIExp ^2^, p=0.003). However, from an analytical perspective, the model’s interpretation and diagnostics still have several critical weaknesses, such as:*

*The authors report that the interaction coefficient (AIExp × SMMS, in Table 3) is not significant, but in their narrative, they occasionally still attempt to link this result as evidence of labor restructuring toward a supervisory economy. This null result actually only indicates that general managerial intensity does not moderate the impact of AI on unemployment.*

**5. Are all the source data underlying the results available to ensure full reproducibility?**

 My choice: **Partly**

**Justification:**

*All raw inputs are sourced from publicly available data. However, since the paper’s main empirical contribution relies on self-constructed panel data (particularly the sector-weighted AI exposure index), replication cannot be performed (straightforwardly) unless the authors upload the cleaned dataset along with the R code to an open repository.*

**6. Are the conclusions drawn adequately supported by the results?**

 My choice: **Partly**

**Justification:**

*The conclusion regarding the concave (inverted-U) relationship between AI exposure and unemployment is well supported by the 2014–2023 panel data. Unemployment was found to increase at low levels of exposure but decrease at higher levels of exposure. However, the conclusion stating that “oversight roles can protect employment” is not supported by the empirical evidence from this model, as the interaction variable is not significant and the proxy used is too broad. The authors should clearly distinguish between purely empirical findings (the AI concave curve) and policy implications of a conceptual/theoretical nature.*

Is the work clearly and accurately presented and does it cite the current literature?

Partly

If applicable, is the statistical analysis and its interpretation appropriate?

Partly

Are all the source data underlying the results available to ensure full reproducibility?

Partly

Is the study design appropriate and is the work technically sound?

Partly

Are the conclusions drawn adequately supported by the results?

Partly

Are sufficient details of methods and analysis provided to allow replication by others?

Partly

Reviewer Expertise:

Data Analytics, Panel Data Modeling,  Artificial intelligence

I confirm that I have read this submission and believe that I have an appropriate level of expertise to confirm that it is of an acceptable scientific standard, however I have significant reservations, as outlined above.

# Reviewer report: Reviewer response for version 2

**DOI:** 10.5256/f1000research.196037.r460646

**Published:** 2026-03-04

**Authors:** Iyanu Emmanuel Olatunbosun

**Affiliations:** 1 University of York, York, UK

The manuscript is clearly presented and addresses a relevant and timely topic. It engages with recent and appropriate literature on AI, automation, and labour market transformation. However, the literature review is somewhat broad and includes discussions not directly linked to the empirical analysis. 

 The study design is appropriate, and the use of panel data and fixed effects modelling is suitable for examining labour market trends across countries. However, the use of managerial employment share as a proxy for AI supervisory roles raises construct validity concerns. 

 The manuscript provides a general methodological overview, but key details required for replication are lacking. The statistical methods used are appropriate for the research questions, and the interpretation is generally reasonable. However, the manuscript occasionally overinterprets associations as structural labour transitions without sufficient causal evidence. 

 The manuscript references secondary data sources, but does not provide sufficient detail on data construction or access to derived variables such as the AI exposure index. This limits full reproducibility. 

 The conclusions are partially supported by the findings, but some claims extend beyond the empirical evidence presented. The authors should present conclusions more cautiously and clearly distinguish between empirical findings and broader theoretical or policy implications.

Is the work clearly and accurately presented and does it cite the current literature?

No

If applicable, is the statistical analysis and its interpretation appropriate?

Partly

Are all the source data underlying the results available to ensure full reproducibility?

Yes

Is the study design appropriate and is the work technically sound?

Yes

Are the conclusions drawn adequately supported by the results?

Yes

Are sufficient details of methods and analysis provided to allow replication by others?

Yes

Reviewer Expertise:

Artificial Intelligence Literacy, Digital Technology, and Finance

I confirm that I have read this submission and believe that I have an appropriate level of expertise to confirm that it is of an acceptable scientific standard.

# Reviewer report: Reviewer response for version 2

**DOI:** 10.5256/f1000research.196037.r458365

**Published:** 2026-02-25

**Authors:** Kwan Hong Tan

**Affiliations:** 1 Singapore University of Social Sciences, Singapore, Singapore

**1) Clear/accurate presentation + current literature**

**Partly**

**Justification:** The manuscript cites a broad and largely current literature, but the presentation is not fully clear because the paper’s central “supervisory economy” mechanism is discussed in AI-specific terms (oversight/governance roles) while the empirical proxy is an economy-wide managerial share. This weak operationalisation blurs what is actually being tested and risks misleading readers about the evidentiary basis for the claims.

**2) Study design appropriate + technically sound**

**Partly**

**Justification:** The econometric design is technically standard and can test an association between AI exposure and unemployment, as well as whether *general managerial intensity* moderates that association. However, it is not well-aligned to the central hypothesis about the emergence of AI-specific oversight labour, because total senior/middle management share is a coarse proxy for AI supervision capacity. As a result, the design is not appropriate for the strongest “supervisory economy” causal claims as framed.

**3) Replication details sufficient**

**Partly**

**Justification:** The manuscript provides the main regression specification and identifies the secondary data sources. However, full replication would require more transparency on (i) the exact construction steps for the AI-exposure index (including transformations and weighting), (ii) treatment of missingness and any imputation rules, and (iii) a clear country-year availability table. Without code and a fully reproducible data-construction pipeline, replication is possible in principle but not straightforward.

**4) Statistical analysis & interpretation appropriate**

**Partly**

**Justification:** The modelling approach (two-way fixed effects with a quadratic term) is a conventional specification for exploring non-linear associations. The main concerns are interpretive and diagnostic: the paper draws strong mechanism-based conclusions from a non-significant interaction using a coarse proxy, and several coefficients (e.g., the very large ln(GDPpc) effect) raise plausibility and scaling/specification concerns that should be explicitly discussed and stress-tested via robustness checks and marginal-effect plots within the observed support.

**5) Source data available for full reproducibility**

**Partly**

**Justification:** The underlying inputs appear to come from public sources (e.g., World Bank/ILO). However, full reproducibility requires access to the exact constructed panel and the complete AI-exposure index construction steps (and ideally code). If these are not deposited as source data/materials, reproducibility remains partial even if all raw inputs are publicly accessible.

**6) Conclusions supported by results**

**No**

**Justification:** Several conclusions are not adequately supported by the results as presented. In particular, claims about the protective role of “oversight roles” are not empirically identified because the proxy measures general managerial share rather than AI-specific oversight labour. Policy recommendations are conceptually interesting but are not tightly linked to the estimated moderation effects. The most clearly supported empirical contribution is the reported non-linear (inverted-U) association between AI exposure and unemployment, which should be separated from stronger supervisory-economy claims.

**Notes to the Authors**

 Thank you for the opportunity to review your thought-provoking paper. The topic is of immense importance and would serve as a timely contribution in view of the current economic climate, with your revisions meaningfully improving the clarity of your arguments. At the same time, please allow me to suggest that several persistent/significant issues be addressed before the paper can be considered for indexing.

**Major Comments**

**Fundamental Mismatch Between Hypothesis and Empirical Test:** This is the most critical issue.

**Your Hypothesis (H2):** “sectors with high AI-adoption intensity... exhibit offsetting growth in supervisory vacancies.” You specifically mention roles like “AI-governance analyst,” “algorithmic-risk manager,” and “prompt engineers” in your introduction and literature review.

**Your Empirical Proxy:** Meanwhile, you use “Employment in senior and middle management (% of total employment)”. This measures the stock of all managers, from a CEO of a manufacturing firm to a head of retail. It does not measure the flow of new, AI-specific supervisory roles.

**Construct mismatch / weak operationalisation of the core mechanism:** The paper’s central “supervisory economy” mechanism concerns the expansion of AI-specific oversight labour (e.g., AI governance, algorithmic risk, model monitoring). However, the empirical proxy (SupEmp/SMMS) captures the stock share of all senior and middle managers economy-wide, most of whom are not engaged in AI oversight. As such, the interaction test between SMMS and AI exposure is a weak operationalisation of the hypothesised mechanism and is unlikely to be diagnostic. A null interaction therefore does not meaningfully adjudicate the supervisory-economy claim; it largely tests whether general managerial intensity cushions unemployment during technological change, which is a weaker and less novel proposition.

**The Consequence:** Therefore, your model would not test your hypothesis. Finding that the total share of managers does not significantly moderate the AI-unemployment relationship tells us nothing about the potential of a supervisory economy built around AI oversight. It is akin to testing whether an increase in the number of all vehicles (cars, trucks, motorcycles) on the road correlates with a decrease in pedestrian injuries to test the safety of a new bike lane network. The data is too coarse to answer the question. The paper would be much stronger if it, instead focused solely on the inverted-U relationship, or if it incorporated more granular vacancy data (like the Australian IVI data mentioned in the introduction) into the core panel model.

**Data and Temporal Limitations:**

 The panel ends in 2023, which the paper acknowledges as the year when generative AI adoption “accelerates at an exponential pace.” This is a critical limitation. The period under analysis (2014-2023) captures the pre-generative AI era and its very early stages. The conclusions, particularly about the turning point (1.7σ above the mean), are based on a technological landscape that no longer exists. The “long-run verdict” remains tentative, but the paper’s conclusions are framed more definitively than this severe data constraint warrants. The policy implications, while exploratory, still feel premature when derived from a pre-2023 world.

**Framing and Overinterpretation:**

**Conclusion:** The statement “AI adoption need not aggravate unemployment when a critical mass of oversight roles is in place” is not supported by your model because you did not measure “oversight roles.” Your model shows that when the total number of managers is high, the relationship between AI and unemployment is unchanged (non-significant interaction). This finding cannot be flipped to support the positive statement you’ve made.

**Title and Core Concept:** Given the empirical findings, the question mark in the title “From Automation-Induced Job Loss to a Supervisory Economy?” is doing a lot of work. Based on your evidence, the answer is a clear “no, at least not as measured by total management share.” The title should be revised to reflect the paper’s actual contribution, such as “The Concave Relationship Between AI Exposure and Unemployment: A Cross-Country Panel Analysis.”

**Policy Implications:** The policy suggestions (e.g., “national AI Oversight Corps,” “Prompt-Engineering upskilling program”) are interesting and logically derived from the conceptual framework. However, they are disconnected from the empirical findings. The evidence suggests that simply having more managers (a very different policy from creating an AI Oversight Corps) doesn’t help. The paper should clarify this disconnect and present these policies as informed by the literature and the concept of a supervisory economy, rather than as a direct implication of the regression results.

**Methodological and Reporting Issues:**

**Control Variables:** The inclusion of R&D expenditure is interesting, but its positive and significant coefficient in the linear model (Table 2) and marginally significant in the quadratic model (Table 3) is interpreted as “short-run displacement.” This could also reflect reverse causality (countries with rising unemployment investing more in R&D as a stimulus) or an omitted variable bias (a third factor driving both high R&D and high unemployment). This interpretation needs to be more cautious.

**Coefficient magnitude check (ln GDPpc):** In Table 3, the coefficient on ln(GDP per capita) is approximately –33.55. Interpreting this literally, a doubling of GDP per capita implies a change of ln(2) ≈ 0.693, which would predict an unemployment change of –33.55 × 0.693 ≈ –23.3 percentage points, an implausibly large within-country effect in most macroeconomic contexts. This suggests potential scaling/measurement issues, strong multicollinearity with fixed effects and controls, or a mis-specified functional form. Please verify variable construction, units, and consider reporting standardized effects or marginal effects within the observed support.

**Table 2 Inconsistency:** Table 2 reports an R² of 0.988, which is suspiciously high for a panel regression on macroeconomic variables, suggesting potential overfitting or a unit root issue.

**Minor Comments**

**Literature Review:** Overall, while very comprehensive, the literature review is overly long and meandering. It could be significantly streamlined to focus more tightly on the core theories (polarization, human capital) and the specific gap the paper aims to fill (the empirical test of the supervisory role). Sections on Indigenous concepts of wealth and dignity, while interesting, feel disconnected from the empirical analysis and could be moved to a discussion section or removed.

**Terminology:** The paper uses “Supervisory Society,” “supervisory economy,” and “AI-supervisory roles” almost interchangeably. While clarified in the introduction, maintaining consistent terminology throughout would improve readability.

**Conclusion**

 To conclude, this paper has a core of interesting and potentially valuable work on the non-linear relationship between AI and unemployment. However, the attempt to link this to a “supervisory economy” is currently unachievable due to a fundamental mismatch between the hypothesis and the empirical proxy used to test it. The over-interpretation of null results and the severe limitations of the dataset further weaken the paper’s conclusions.

 A major revision that either reframes the paper’s contribution around the inverted-U finding or incorporates more precise data to test the core hypothesis is necessary before this work can be considered for indexing.

 Thank you for reading and I look forward to reading your upcoming work.

Is the work clearly and accurately presented and does it cite the current literature?

Partly

If applicable, is the statistical analysis and its interpretation appropriate?

Partly

Are all the source data underlying the results available to ensure full reproducibility?

Partly

Is the study design appropriate and is the work technically sound?

Partly

Are the conclusions drawn adequately supported by the results?

No

Are sufficient details of methods and analysis provided to allow replication by others?

Partly

Reviewer Expertise:

Economics, AI

I confirm that I have read this submission and believe that I have an appropriate level of expertise to state that I do not consider it to be of an acceptable scientific standard, for reasons outlined above.

# Reviewer report: Reviewer response for version 1

**DOI:** 10.5256/f1000research.185698.r438831

**Published:** 2026-01-09

**Authors:** Lukasz Swiatek

**Affiliations:** 1 The University of New South Wales, Australia, Australia

This article provides a timely, rich analysis of the international AI-driven displacement of labour and the growth of supervisory roles. It thoughtfully posits the consequent rise of the ‘supervisory society’ and explores its implications for workers, organisations and policymakers. It can be suggested that the article’s three hypotheses don’t align tightly enough with the current definition of the supervisory society, but this suggestion is debatable and doesn’t thwart the overall premise of the article. The development of a real-time ‘AI Jobs Barometer’ is also helpfully proposed. Nevertheless, various enhancements would strengthen the article. These enhancements are divided, below, into major and minor recommendations.

 Major recommendations

 (1) The notion of the ‘supervisory society’ is thoughtful, though slightly overblown, given the unlikelihood of societies coming to be preoccupied with the supervision of AI. To make this thoughtful idea more realistic, it would be worth modifying it slightly to something like the ‘supervision-focused society’ or the ‘supervision-oriented society’.

 (2) The definition of the ‘supervisory society’ is interesting – “a labour-market equilibrium in which humans specialise in oversight tasks that AI cannot internalise” – but it needs to be strengthened. In particular, it would be excellent for the definition to take into account the nature of society more effectively.

 (3) As the ‘supervisory economy’ and ‘supervisory society’ have already been mentioned briefly in the literature (see https://doi.org/10.1007/978-3-031-32822-0_18 and https://doi.org/10.1007/978-3-319-73888-8_35), it would be worth citing these outputs. It would also be beneficial for the authors to note briefly how their understanding of the ‘supervisory economy’ and ‘supervisory society’ differs from the versions in the literature.

 (4) The observation could be made that the oversight of AI involves more than just supervision. It would be wonderful for the authors to note this point briefly (for example, in the introduction) in order to help make the critical dimension of the article even stronger.

 (5) The paragraph beginning with “Equipping the next generation…” provides a range of interesting educational approaches for higher education institutions, but it currently lacks much-needed ethics-focused elements. It would be excellent for the authors to add at least one teaching and learning element related to AI ethics.

 (6) The Conclusion, and especially the “Policy implications” sub-section, helpfully mention some of the AI issues that need to be considered, though it would be good to note others, even briefly (i.e., even in a sentence or two), given their importance. A major source like Kate Crawford’s ‘Atlas of AI’ (https://doi.org/10.2307/j.ctv1ghv45t) might be helpful in this respect.

 Minor recommendations

 (7) It would be helpful to provide a reference for “Recent diffusion curves show…” or, alternatively, to indicate (for example, by using a phrase like “as noted in section x”) where the relevant data can be found.

 (8) The suggestions relating to the “First Step Student Program” are very useful, though at least one reference is needed in that paragraph to help readers obtain further information (if not about the First Step Student Program itself, then about Australia and its tertiary education sector).

 Overall, the article and its supervision-related insights make useful contributions to the AI scholarship. The implementation of the above-listed enhancements would help make an already-valuable and well-written article even stronger.

Is the work clearly and accurately presented and does it cite the current literature?

Partly

If applicable, is the statistical analysis and its interpretation appropriate?

I cannot comment. A qualified statistician is required.

Are all the source data underlying the results available to ensure full reproducibility?

Yes

Is the study design appropriate and is the work technically sound?

Yes

Are the conclusions drawn adequately supported by the results?

Yes

Are sufficient details of methods and analysis provided to allow replication by others?

Yes

Reviewer Expertise:

Artificial intelligence, extended intelligence, other technological developments (especially in relation to media and communication)

I confirm that I have read this submission and believe that I have an appropriate level of expertise to confirm that it is of an acceptable scientific standard, however I have significant reservations, as outlined above.

# Response

**Published:** 2026-01-31

**Authors:** William Pacheco

**Affiliations:** Economics, University Anahuac Mexico, Huixquilucan de Degollado, State of Mexico, Mexico

We thank the reviewer for the careful reading of the manuscript and for the constructive and detailed comments. In response, we have clarified the conceptual scope of the “supervisory economy,” strengthened the theoretical justification for non-linear AI effects, refined the econometric specification and diagnostics, and improved transparency regarding variable construction and identification strategy. We have also tightened the link between the empirical results and the policy discussion, explicitly acknowledging limitations where effects are statistically weak. All changes are clearly marked in the revised manuscript and referenced below point by point.

# Reviewer report: Reviewer response for version 1

**DOI:** 10.5256/f1000research.185698.r445493

**Published:** 2026-01-08

**Authors:** Derrick Mirindi

**Affiliations:** 1 School of Architecture and Planning, Morgan State University, Baltimore, Maryland, USA

1. This manuscript requires a full English edition revision. Most importantly, the authors did not properly define all the abbreviations and often missed the definitions.

 2. All the data claims to use from an organization should be cited.

 3. There are too many em dash "—" symbols; please revise. In fact, if you used an AI tool to produce this study, you have to claim it. This listing not only includes texts but also codes used in R.

 4. Revise all subtitles and ensure that they have the same police type, size, and color.

 5. Provide an Excel or Word document containing the **datasets** used in R to plot the figures. 

 6. Include a flowchart figure illustrating this study.

 7. This sentence is long and cannot be a single paragraph: "As breakthroughs in artificial intelligence ... lag the technological frontier." Please, revise.

 8. Revise all the equation formatting, for instance: "β1/(2β2) ≃1.7-\beta_{1}/(2\beta_{2}) \simeq 1.7 β1/(2β2) ≃ 1.7". Also, why are some in bold while others are not?

 9. The literature review section is too long. If possible, can you reduce it?

Is the work clearly and accurately presented and does it cite the current literature?

Yes

If applicable, is the statistical analysis and its interpretation appropriate?

Partly

Are all the source data underlying the results available to ensure full reproducibility?

No

Is the study design appropriate and is the work technically sound?

Yes

Are the conclusions drawn adequately supported by the results?

Partly

Are sufficient details of methods and analysis provided to allow replication by others?

Partly

Reviewer Expertise:

Artificial intelligence, construction, architecture, wood, automation, deep learning

I confirm that I have read this submission and believe that I have an appropriate level of expertise to state that I do not consider it to be of an acceptable scientific standard, for reasons outlined above.
